# Dynamics of HIV-1 Assembly and Release

**DOI:** 10.1371/journal.ppat.1000652

**Published:** 2009-11-06

**Authors:** Sergey Ivanchenko, William J. Godinez, Marko Lampe, Hans-Georg Kräusslich, Roland Eils, Karl Rohr, Christoph Bräuchle, Barbara Müller, Don C. Lamb

**Affiliations:** 1 Physical Chemistry, Department of Chemistry and Biochemistry, Munich Center for Integrated Protein Science (CiPSM) and Center for NanoScience, Ludwig-Maximilians-Universität München, Munich, Germany; 2 University of Heidelberg, BIOQUANT, IPMB, and German Cancer Research Center (DKFZ), Department of Bioinformatics and Functional Genomics, Biomedical Computer Vision Group, Heidelberg, Germany; 3 Department of Virology, Universitätsklinikum Heidelberg, Heidelberg, Germany; 4 Department of Physics, University of Illinois at Urbana-Champaign, Urbana, Illinois, United States of America; Yale University School of Medicine, United States of America

## Abstract

Assembly and release of human immunodeficiency virus (HIV) occur at the plasma membrane of infected cells and are driven by the Gag polyprotein. Previous studies analyzed viral morphogenesis using biochemical methods and static images, while dynamic and kinetic information has been lacking until very recently. Using a combination of wide-field and total internal reflection fluorescence microscopy, we have investigated the assembly and release of fluorescently labeled HIV-1 at the plasma membrane of living cells with high time resolution. Gag assembled into discrete clusters corresponding to single virions. Formation of multiple particles from the same site was rarely observed. Using a photoconvertible fluorescent protein fused to Gag, we determined that assembly was nucleated preferentially by Gag molecules that had recently attached to the plasma membrane or arrived directly from the cytosol. Both membrane-bound and cytosol derived Gag polyproteins contributed to the growing bud. After their initial appearance, assembly sites accumulated at the plasma membrane of individual cells over 1–2 hours. Assembly kinetics were rapid: the number of Gag molecules at a budding site increased, following a saturating exponential with a rate constant of ∼5×10^−3^ s^−1^, corresponding to 8–9 min for 90% completion of assembly for a single virion. Release of extracellular particles was observed at ∼1,500±700 s after the onset of assembly. The ability of the virus to recruit components of the cellular ESCRT machinery or to undergo proteolytic maturation, or the absence of Vpu did not significantly alter the assembly kinetics.

## Author Summary

Human immunodeficiency virus (HIV) particles are formed and released at the plasma membrane of the infected cell. Here, we analyzed the dynamics of HIV assembly and release making use of fluorescently labeled HIV structural proteins. We determined that assembly of the viral protein shell occurs within ∼8–9 min after nucleation of an assembly site and virus particles are formed individually and not from large patches. Virion release was observed ∼25 min after nucleation of the assembly site. Assembly of the Gag shell thus appears to constitute only a minor part of the period required for particle formation indicating that traversing the membrane and fission are the rate-limiting stages in virion formation. Using a photoconvertible label in the viral Gag protein, we established that the Gag molecules driving nucleation of a new assembly site and in bud growth are recruited preferentially from the cytosolic pool of Gag molecules and from recently membrane-attached Gag. No intracellular assembly or vesicular trafficking of Gag was observed. The described results add essential dynamic information to our picture of virus release and provide an experimental basis for interfering with this stage of virus replication.

## Introduction

Assembly and release of progeny virions are fundamental steps in viral replication. In the case of retroviruses, such as human immunodeficiency virus type 1 (HIV-1), the viral structural polyprotein Gag plays a central role in mediating both of these processes which occur concomitantly at the plasma membrane of the infected cell. Gag comprises domains required for membrane binding, multimerization, nucleic acid binding as well as for interaction with the host cell derived budding machinery and has been demonstrated to direct the formation of virus like particles (VLP) in the absence of other viral proteins [1]. During or shortly after virus release, Gag is cleaved by the viral protease resulting in domain separation and maturation of the infectious virion (for review see [2],[3],[4]).

The assembly process, leading from monomeric Gag molecules translated at cytoplasmic polysomes to the virus bud comprising several thousand Gag molecules at the plasma membrane, has been investigated using a variety of techniques. Results from pulse-chase labeling, density gradient fractionation, time-lapse fluorescence imaging and intracellular fluorescence resonance energy transfer measurements have resulted in a current general view of retroviral assembly: Gag is rapidly converted from a soluble form to a multimeric, membrane associated complex and this process is reflected by changes in the distribution of Gag within the cell (e.g. [5],[6],[7],[8],[9],[10]). Initial Gag-Gag interactions occur prior to arrival at the plasma membrane, while assembly of higher order structures appears to be confined to the plasma membrane. At the membrane, Gag localizes to or induces membrane microdomains from which budding and release are believed to occur (e.g. [11],[12],[13],[14]). All these steps may be influenced by cellular factors, but these factors and their mechanistic role are currently poorly understood (reviewed in [2],[15]). Completion of budding and release require the cellular ESCRT machinery, normally involved in endosomal sorting and cytokinesis (reviewed in [16],[17],[18]).

While the general pathway of HIV morphogenesis has been elucidated in recent years, several aspects are still controversial and the details of this process and in particular its kinetics are poorly understood. Oligo- or multimers of Gag have been detected in the cytoplasm and at intracellular membranes [10],[19] and are believed to be assembly intermediates, but the size of Gag complexes added to a growing bud and their route of trafficking are currently not known. It is also unclear, whether Gag molecules are recruited from the cytoplasm or an intracellular membrane to the budding site or whether they first associate with the plasma membrane and are then organized into a budding structure by lateral movement. Budding may occur from platforms giving rise to multiple consecutive budding events, as has been reported for Rous sarcoma virus [20], or may occur mostly from unique sites, as suggested for Moloney murine leukemia virus [21]. Finally, the kinetics of assembly have only recently been described [22] and kinetics of release are unknown at present. While traditional biochemical and cell biology techniques are not well suited to address these aspects, insight can be gained from direct visualization of budding site formation at the plasma membrane with high time resolution. Such studies are now possible due to recent advances in technology. Gladnikoff et al. applied atomic force microscopy to monitor retroviral budding [21], albeit with a technically limited time resolution of several minutes per frame. Atomic force microscopy is only suited for the analysis of budding, however, since it measures distortion of the membrane, while analysis of the initial nucleation complex and early assembly requires other techniques.

To investigate the kinetics of HIV assembly at high time resolution, we employed a combination of wide-field (WF) and total internal reflection fluorescence (TIRF) microscopy in conjunction with fluorescently labeled and photoconvertible HIV-1 derivatives. Jouvenet et al. also analyzed the dynamics of VLP formation by fluorescently labeled Gag polyproteins and reported assembly of Gag complexes to occur within minutes [22]. Here, we confirm and extend this analysis by investigating the dynamics of HIV-1 assembly in more detail, using a complete viral plasmid expressing all HIV-1 proteins except Nef and determining the time until a complete virion is released. Virions assembled at individual sites and rarely appeared to bud from larger Gag platforms. The rate of assembly was similar for wild-type HIV and for variants that lack the PTAP late domain motif or are deficient in protease activity. Using a recently developed photoconvertible protein, it was found that nucleation of the assembly site and bud growth occurred preferentially from cytosolic Gag molecules. The average time from the appearance of an assembly site at the plasma membrane to release was 1,500±700 seconds.

## Results/Discussion

### Rapid accumulation of HIV-1 assembly sites after initial nucleation

The HIV-1 Gag protein is the main driving force for virion assembly and Gag by itself can assemble into virus-like particles which are released from expressing cells [1]. However, the details of the assembly and release processes may be influenced by other virus components. Furthermore, it has been demonstrated that the steps of Gag targeting and release may be influenced by the nuclear export pathway of its encoding mRNA, which in the case of HIV is mediated by the virus encoded protein Rev [23]. For this reason, we believe that Gag analyzed in a viral context will most accurately reflect the kinetics of HIV-1 assembly and release in living cells. Thus, we made use of a fluorescently labeled HIV-1 derivative with the *egfp* gene inserted between the MA and CA coding regions of the *gag* gene. Cells transfected with an equimolar mixture of this construct and its wild-type (wt) counterpart produce highly fluorescent particles displaying wt morphology and infectivity [24]. For the microscopic analyses described here, non-infectious derivatives lacking the long terminal repeat regions [25] were used. These constructs express Gag in the viral context, i.e. in the presence of all other HIV proteins (except for Nef) and dependent on Rev mediated nuclear export of the encoding RNA. For these reasons, Gag accumulation is slower and overall Gag expression levels are much lower than for the Rev-independent, codon-optimized version of Gag alone used in a previous study [22]. For our studies, time points between 20 and 30 h post transfection (hpt) were found to be optimal for microscopic observation of budding site formation in HeLa cells.

**Figure 1:**
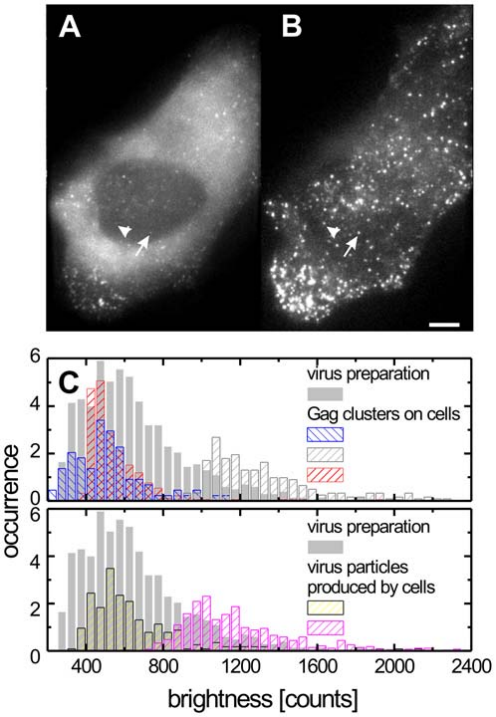
Live cell imaging of individual HIV-1 assembly sites. HeLa cell transfected with a mixture of pCHIV and pCHIV^eGFP^ imaged at 25 h hpt (A) in WF and (B) in TIRF mode. The scale bar represents 5 µm. The arrow and the arrow head indicate the two individual punctae analyzed in Figure 2A and B respectively. (C), Normalized histograms of fluorescence intensities from Gag punctae on the cell surface (upper panel, shown in hatched bars with different colors representing different cells) and released particles that adhered to the coverslip after cell retraction during a live-cell imaging experiment (*lower panel*, *hatched bars*). For comparison, the fluorescence intensity distribution of a purified particle preparation is shown in grey bars in both panels (the normalized area of the histogram has been scaled by a factor of 2 for clarity).

Jouvenet et al. have demonstrated the capability of investigating the assembly of HIV-1 with TIRFM [22]. In order to gain quantitative information over the assembly process, it is necessary to distinguish between changes in the fluorescence intensity arising from the incorporation of new fluorescently labeled Gag particles and axial movements of the complex in the exponentially decaying evanescent field of the TIRF microscope [26]. To this end, we have constructed a custom-made microscope capable of switching between TIRF and WF microscopy modes in alternating frames up to frame rates of 30 Hz. A diagram of the experimental setup is shown in Figure S1 and details can be found in the materials and methods and supporting information. Figure 1A and B shows a cell producing fluorescent HIV-1 imaged by WF and TIRF microscopy, respectively. Individual punctate clusters of Gag.eGFP were detected and tracked in TIRF mode and the corresponding fluorescence of the punctae in WF was determined. As can be seen in Video S1, the fluorescent punctae were typically mobile. In order to perform a quantitative, statistically meaningful analysis, software was developed to semi-automatically track large numbers of individual punctae in up to three different color channels or different microscopy modes. The background-corrected intensity, particle location, and instantaneous velocities of the individual assembly complexes were calculated and analyzed (see also SI and ref [27]).

In the time window of 20–30 hpt chosen for our analysis, the extent of Gag expression varied strongly from cell to cell. Some cells were already displaying bright fluorescence at 20 hpt while others showed detectable fluorescent Gag expression only towards the end of the observation period. The appearance of Gag punctae in our system did not correlate with the level of Gag expression in contrast to the results obtained for Gag.GFP in the previous study by Jouvenet et al [22] who reported a gradual change in the rate of assembly of Gag complexes with time. This difference may be due to the faster and higher expression of this codon-optimized Gag encoding construct used in the previous study and possibly also to its Rev-independence. Some cells exhibited bright cytosolic fluorescence over several hours without showing formation of punctate clusters. Hence, we selected cells where the first punctae at the plasma membrane became detectable at the beginning of the observation period and measured the dynamics of assembly and budding over 1 to 2 hours with a temporal resolution of 2 s/frame. Punctate clusters rapidly accumulated at the plasma membrane within approximately one hour after the initial detection of individual nucleation sites (Figure S2). At later time points, the cell membrane became densely packed with punctate clusters, which made tracking of individual clusters problematic. Cotransfection of constructs expressing Gag.mCherry and the early endosome marker GFP.Rab5 revealed no colocalization of Gag.mCherry punctae with early endosomes during our observation window (data not shown). This is consistent with the fact that the constructs used express the viral protein Vpu, which is able to counteract attachment and endocytosis of newly produced virions proposed to be mediated by cellular restriction factors in HeLa cells [28],[29],[30].

### Assembly sites correspond to single nascent virions

To determine whether the observed punctae represent individual nascent virions or larger accumulations of Gag molecules serving as budding platforms, we estimated the overall size of Gag.eGFP punctae from their relative fluorescence intensity. The fluorescence intensity distribution of punctate assembly sites was compared to that of particles released during the live-cell imaging experiments, which were found attached to the cover slip between HIV-1 producing cells, as well as to individual particles from a purified HIV^eGFP^ preparation (Figure 1C). We have previously established that purified HIV^eGFP^ preparations consist of a single particle population with an average diameter of 172±10 nm and do not contain large aggregates [31]. However, the fluorescence intensities of individual particles are broadly distributed corresponding to the variability of particle diameters and total number of Gag molecules per virion [32],[33]. Recent results further suggest that Gag stoichiometry also varies with the amount of Gag produced per cell [33]. Furthermore, the co-expression system used in this study can result in variations in the ratio of labelled to unlabelled Gag between different cells. Accordingly, differences in average particle intensities between individual cells as well as between experiments were observed. However, the fluorescence intensity of membrane associated Gag clusters was consistently less than or similar to that of free particles bound to the coverslip during the course of the imaging experiment or of particles purified from the supernatant of virus producing cells (Figure 1C). This suggests that the Gag.eGFP assemblies in the stationary phase (phase II discussed in the next section) contain roughly the same amount of Gag as individual released particles and do not represent larger assembly patches but rather individual virus buds.

This conclusion is supported by the observation that appearance of multiple particles from the same site was very rare. Video S2 demonstrates the one instance in several hundred events analyzed where multiple particles appeared to bud from a single site. Similar results have been reported for Rous sarcoma virus using two-photon live-cell imaging [20] and for Moloney murine leukemia virus using atomic force microscopy [21]. Recently, Manley *et al.* measured the movement of HIV-1 Gag fused to a tandem dimer of EosFP using photoactivated localization microscopy [34] and reported that immobile Gag molecules were often found in clusters with a radius of less than 300 nm, which also argues against the formation of large Gag assemblies serving as budding platforms.

### Kinetics of virus assembly

**Figure 2:**
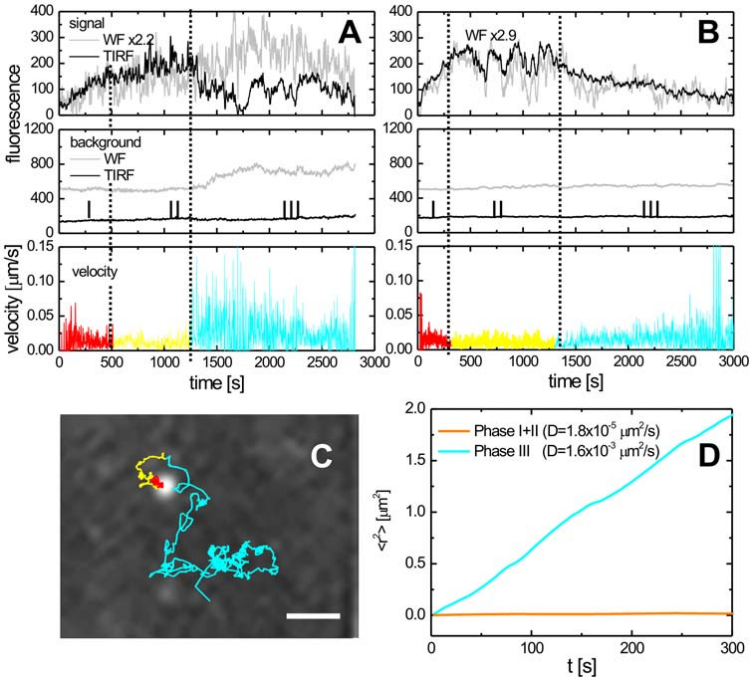
The three phases of HIV-1 assembly. (A–B) Intensity traces of two individual Gag.eGFP clusters indicated with an arrow (A) and arrow head (B) in Figure 1 recorded in TIRF (black line) and WF (grey line) modes are shown in the *top panels*. Three phases, separated here by dashed lines, can be observed: Phase I) a rapid rise in fluorescence intensity, Phase II) a plateau region with fluctuations in fluorescence intensity, and Phase III) a decay of the fluorescence signal. The *middle panels* show the intensity traces of the local background. The *bottom panel* displays a plot of the corresponding instantaneous velocities of the particles. In A, an abrupt increase in instantaneous velocity is observed concomitantly with the onset of Phase III. (C) Trajectory of the individual Gag.eGFP cluster from panel A color-coded according to the three different phases. The scale bar represents 1 µm. (D) A mean-square displacement analysis of the trajectory shown in panel C indicates the random character of motion in the different phases and shows a change by two orders of magnitude in diffusion coefficient between the phases I+II and III.

Having established that Gag clusters correspond to individual assembly sites, we investigated the kinetics of the assembly process. Figure 2A and B display the appearance of two individual fluorescent clusters (indicated by arrows in Figure 1A and B) of Gag.eGFP above the cytosolic background. Fluorescence intensities of individual clusters were found to change in three characteristic phases. Formation of the budding site was observed as a rapid increase in fluorescence intensity during the first few minutes after initial detection (Phase I), followed by a stationary phase where the fluorescence signal fluctuates about a constant value (Phase II). The duration of this plateau phase varied between individual clusters. Subsequently, a decay of fluorescence intensity was observed (Phase III).

**Figure 3:**
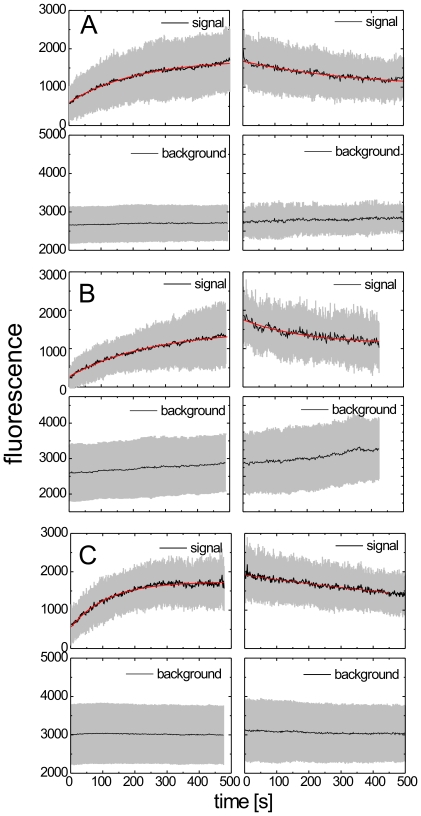
Average kinetics of phases I and III. The rates of processes involved in HIV^eGFP^ assembly determined from single cells using TIRF microscopy. Shown are the averaged data (black line), the standard deviation of the individual traces (grey halo) and the fit of the average data to a saturating (Phase I, *left panels*) or decaying (Phase III, *right panels*) exponential (red line). Data are shown for 125 traces from a cell expressing wt HIV-1 (A), 89 traces from a ESCRT-recruitment defective variant displaying diminished release (B) and 69 traces from a maturation defective variant carrying a mutation in the PR active site (C). As the measurements were performed with different camera settings, the fluorescence intensities have been normalized to allow a direct comparison between experiments.

**Figure 4:**
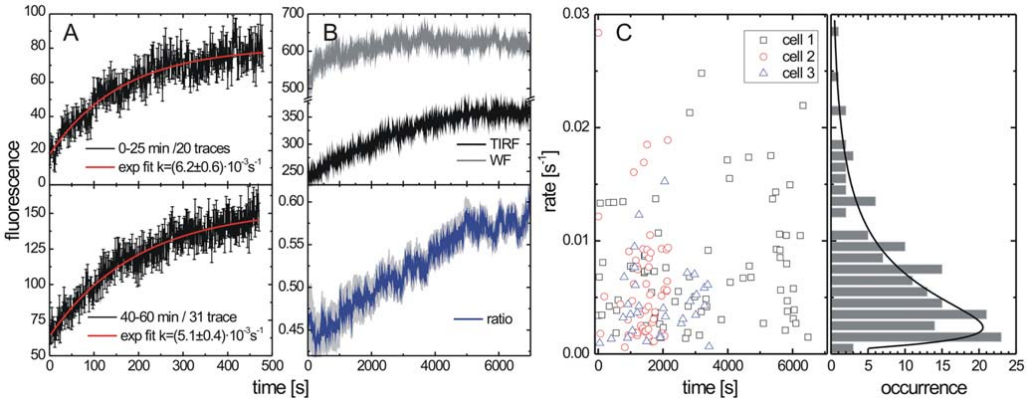
Rates of Gag assembly versus time. Assembly rates were measured at different time intervals after appearance of the first assembly sites. (A) Average fluorescence intensity traces of clusters which appeared between 0 and 25 minutes (*upper panel*) and 40–60 minutes (*lower panel*) after the onset of virus production on the surface of a single cell. (B) background in WF and TIRF channels (*upper panel*) indicating the concentration of Gag in the cytosol and plasma membrane respectively and ratio of the fluorescent background in TIRF and WF (*lower panel*). The grey halo in the lower panel shows the standard deviation of the data. (C) Rates of assembly versus time of first detection for three individual cells (*left panel*) and histogram summarizing all rates from the same three cells (*right panel*). The black line shows a log-normal fit yielding an average rate of (5.5±0.5)×10^−3^ s^−1^.

During phase I, three types of kinetic behavior were observed: a saturating exponential (in ∼80% of all cases), a linear increase in intensity until saturation was reached (10–20%) or intensities that increased exponentially until reaching a plateau (<3%) (Figure S3). Here, we concentrate on the most prevalent behavior, the saturating exponential. To determine the kinetics of assembly, we averaged 309 individual traces with a minimal length of 300 frames, corresponding to 10 min (Figure 3A). The asynchronous initiation of assembly sites was compensated for by aligning the intensity traces before averaging. Details of the averaging process are given in the Supporting Text S1. The average value (black line) and the standard deviation (light grey halo) of the selected traces are plotted for measurements on single cells. In contrast to Jouvenet et al. [22], who measured the time between the onset of assembly and saturation, we describe the budding kinetics in terms of a model function. The red line displays the fit of the data to a saturating exponential function, 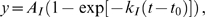
(1)where *A_I_* is the maximum fluorescence amplitude, *k_I_* is the rate of fluorescence increase and *t*_0_ is the time when assembly begins. By averaging and fitting the kinetics to a model function, our estimation of the assembly rate is independent of uncertainties in detection of the particle at early time points and in the subjective determination of when assembly is complete. The values derived from experiments on multiple cells are summarized in Table 1. The rate of assembly is ∼4.3×10^−3^ s^−1^ or 233 s, which corresponds to a time of ∼9 minutes for 90% completion of assembly. To determine the distribution of assembly rates for individual sites, single traces were fit separately to equation (1). The resulting histogram (Figure 4C, left panel) could be approximated by a log-normal distribution of rates. The maximum position of (5.5±0.5)×10^−3^ s^−1^ obtained from this distribution corresponds well to the rate of 4.3×10^−3^ s^−1^ determined from the averaged traces shown in Figure 3A.

**Table 1:** Kinetic rates of Gag assembly for phase I and phase III.

Gag variants	Phase I (N)	Rate Phase I *k_I_* (s^−1^)	Phase III (N)	Rate Phase III *k_III_*(s^−1^)
WT	309	(4.3±0.5)×10^−3^	49	(2.7±0.2)×10^−3^
Late -	191	(5.5±1.8)×10^−3^	24	(5.2±0.9)×10^−3^
PR -	184	(7.9±1.5)×10^−3^	47	(2.2±0.4)×10^−3^
ΔVpu	119	(4.6±1.5)×10^−3^		
WT (SDCM)	60	(5.5±0.3)×10^−3^		*

*: Not Determined.

Kinetic rate constants were determined from data collected using TIRFM or SDCM. The errors are the standard deviation calculated from measurements on multiple cells (4 cells for WT and PR- , 3 cells for the Late- variant and 2 cells for ΔVpu) and include the cell to cell variation in the rate of assembly. For measurements using SDCM, the error represents the standard error of the exponential fit.

We confirmed the observed assembly rates using SDCM (Video S3). While SDCM does not offer the contrast of TIRF microscopy, it has the advantage that three-dimensional information is collected and a projection of the z-stack can then be used for tracking of the individual assembly sites. Hence, the measured fluorescence intensity is not sensitive to axial motion of a cluster over several micrometers. The SDCM measurements confirmed the saturating exponential kinetics of Phase I yielding comparable rates (*k_I_* = 5.5×10^−3^ s^−1^ for the ensemble average in Figure S4 and *k_I_* = 3.6×10^−3^ s^−1^ for the typical trace shown in Figure S4A). The observed characteristic time of ∼200 s (or ∼8–9 min for 90% completion of assembly) is in a similar range with the estimate of 5–6 minutes for complete assembly made by Jouvenet et al [22].

The variation of rates between individual sites on one cell suggests that additional factors besides the overall Gag concentration influence the rate of assembly. We analyzed the rate of HIV assembly at early (0–25 minutes) and intermediate (40–60 minutes) time points of Video S1. Between these time intervals, the concentration of Gag at the plasma membrane increased significantly whereas the concentration of Gag in the cytosol remained relatively constant. The assembly rates for the two time periods were similar (compare Figure 4A and 4B). Moreover, we observed no change in the assembly rates of individual assembly sites as a function of time after onset of punctae formation (Figure 4C). Considering that some cells exhibited cytosolic fluorescence for hours without producing virus particles and that the rate of assembly appear to be independent of Gag concentration one can speculate that additional factors are involved in the initiation of assembly with some cells apparently lacking assembly competence.

The observed saturating exponential increase in fluorescence intensity indicates that Gag accumulation at a growing budding site is retarded over time and eventually stops. The simplest explanation for this observation is that completion of the spherical Gag shell is preventing the addition of further Gag molecules. However, this is inconsistent with data from electron cryo-tomography experiments showing that the Gag shell of immature particles is incomplete [35],[36]. Therefore, other mechanisms must be responsible for restricting Gag incorporation, such as Gag-induced membrane curvature or closure of the bud neck by the cellular ESCRT machinery restricting access for further Gag molecules.

After the initial increase in fluorescence intensity (phase I), the signal fluctuated about a nearly constant average intensity for variable time periods (Phase II) before a decay in fluorescence intensity was observed (phase III). Using photobleaching experiments, Jouvenet et al. [22] have shown that there is no exchange of Gag between the assembly site and the cellular environment during the plateau region following the assembly phase. This phase most likely reflects the interaction of the assembled Gag shell with cellular membrane constituents but further investigations are required to elucidate the biological significance of phase II. We determined the duration of phases I+II, i.e. the time period between the first detection of the assembly site and the onset of phase III, for 160 individual trajectories (Figure S5A). The total duration of phase I+II is 1,300±700 s. Using 90% saturation of phase I (90% level of the exponential amplitude in equation (1)) as the onset of the phase II, the average duration of phase II was determined to be ∼10 min. The duration of phase II varied between individual events and the distribution is shown in Figure S5B.

Phase III is defined by a decay in fluorescence intensity of the TIRF signal. It began abruptly as seen in Figure 2A and 2B, indicating a change in particle behavior. This phase was also frequently observed in wide-field mode. We characterized the dynamics of phase III by averaging multiple traces as we did to analyze phase I (see Supporting Text S1). The results were fit to a decaying exponential function with an offset: 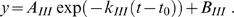
(2)The results are given in Table 1. A rate of 2.7×10^−3^ s^−1^ was determined for phase III. Many physical processes can potentially contribute to the decay of fluorescence intensity. These processes include (i) loss of Gag.eGFP molecules from the assembly site, (ii) a change in the distance of the particle from the cover slip or (iii) increased motility of the particle upon budding or dissociation from cellular factors. Most likely, multiple factors are responsible for the fluorescence decay in phase III and their contribution may vary from event to event. For example, formation of the budding structure may cause Gag molecules to approach the coverslip. This would lead to higher illumination intensities and faster photobleaching rates. Hence, a short rise in fluorescence intensity would be observed at the end of phase II followed by the decrease in fluorescence intensity characteristic of phase III. Such fluorescence intensity curves were occasionally observed for specific assembly sites but were in general not indicative of the transition from phase II to phase III. In other cases, we observed an increase in mobility of the particle (as discussed below). When the position of a particle changes during the integration time of the camera, the signal becomes more diffuse leading to a decrease in peak intensity. Currently, we cannot determine what physical process or processes are, in general, responsible for Phase III. However, the results suggest that the onset of phase III is related to formation of late budding structures and/or the budding event. A detailed analysis of single traces provided a clear indication for virus release at the onset of phase III in individual cases.

### Virus release

Release of HIV-1 from the producer cell should be accompanied by an increase in mobility of the virion. The particle depicted in Figure 2A, underwent a dramatic increase in its instantaneous velocity concomitantly with the onset of Phase III. The trajectory of the particle, color coded for the three phases is shown in Figure 2C and a movie of the assembly and release of the particle is shown in the Video S4. A mean-square-displacement analysis of the trajectory starting at Phase III shows random Brownian motion with a 100 fold increase in the diffusion coefficient (Figure 2D). The mobility of the particle is significantly higher than expected for the movement of HIV-1 within the cytosol in the absence of directed transport, although still lower than the diffusion coefficient of HIV-1 in buffer alone [31]. The diminished diffusion coefficient is likely due to confinement of the virus between the cover slip and the cell.

Such clear signatures of release were rare. Due to the TIRF geometry, we can only observe particles budding towards the cover slip. These virions are typically constricted in the narrow space between the cell and the cover slip or may even adhere nonspecifically to the glass surface. In addition, released viruses may be tethered to the plasma membrane upon release by interaction with cell surface constituents. Complete assembly and release could be tracked in TIRFM for a total of 18 particles starting from nucleation of the assembly site and using changes in diffusional behavior or rapid disappearance of the particle as a marker for release. Of these 18 events, 5 disappeared within the first 60 seconds after the onset of Phase III and 8 trajectories showed anomalies in the MSD analysis due to sticking of the virus for a few frames to the glass cover slip and/or cell surface. Of the remaining 5 traces, all showed random diffusional motion with a diffusion coefficient above 10^−3^ µm^2^/s. From these data, the mean time from appearance of an assembly site to particle release was determined to be 1,500±700 s. This time frame is similar to the time of 1,300±700 s that we had previously determined as the onset of phase III and further supports the idea that the onset of phase III is related to the formation of late budding structures and/or release.

To investigate whether the long delay between assembly and release was influenced by adherence of the ventral cell surface to the coverslip, individual HIV-1 budding events were also recorded on the dorsal membrane using SDCM. In 12 observed events, the average time from initiation of assembly to release was 1,700±1,000 s, indistinguishable from the results obtained with TIRFM. An example of an HIV-1 assembly/release event recorded by SDCM is shown in Figure S6 and Video S5. Besides these clear release events, many more viruses were found to remain associated with the dorsal plasma membrane for extended periods of time (at least 3 hours). This observation is consistent with that of Larson et al. [20] and is probably a result of non-specific sticking of newly produced virions to the cell surface.

Our data indicate that assembly of the Gag shell in HeLa cells occurs with a characteristic time of ∼200 s (or 8–9 min for 90% completion of assembly) followed by an apparent delay of ∼15 min (phase II) before the virion is released. This lag period after formation of the Gag shell was unexpected and further analyses are required to analyze whether release kinetics are similar in different host cells and experimental systems. One may speculate that the delay period serves to complete membrane closure and fission involving host cell functions. It may also be advantageous for the virus. For example, the time lag may allow movement of the bud towards a virological synapse [37], which serves as preferred pathway of virus transmission from cell to cell.

### Defective virus variants

To obtain further insight into what determines the kinetics of assembly, we performed experiments using HIV-1 variants impaired in release due to mutation of the PTAP motif in the p6 domain of Gag [38] or in proteolytic maturation due to a D25A mutation in the PR active site [39]. The results are summarized in Figure 3 and Table 1 and showed no significant differences for the various constructs. The largest difference in the average rates of Phase I was observed between wt and PR-defective constructs. We compared, therefore, the distributions of rates for these two variants. The maximum of the distribution for the PR- variant was at (8.4±0.7)×10^−3^ s^−1^, which compares well with the average value of (7.9±1.5)×10^−3^ s^−1^ from Table 1. Although these values are higher than those obtained for wt (5.5×10^−3^ s^−1^ and 4.3×10^−3^ s^−1^, respectively), statistical evaluation yielded a p-value of 0.74, indicating that the difference is not significant. We conclude that the assembly process corresponding to Phase I is independent of ESCRT recruitment or protease activity. PR-defective HIV-1 had exhibited a decreased rate of release in previous pulse-chase experiments [40], but showed no apparent difference in the assembly phase of individual particles. To obtain further insight, we also analyzed a variant defective in the viral protein Vpu, which is required to counteract the tetherin-mediated restriction to virus release in HeLa cells [28],[41]. Neither the rate of 4.6×10^−3^ s^−1^ for Phase I alone (Table 1), nor the combined duration of Phases I+II of 1,600±1,000 s (Figure S5C) appeared to be significantly different from those observed for the wild-type virus. The average duration of the Phase II (Figure S5D), determined as previously discussed, was calculated to be approximately 10 min, also comparable to the wild type construct (Figure S5B). These observations are consistent with the fact that the tetherin-mediated restriction manifests itself after the actual budding event.

In addition to similar assembly rates and durations of phase II, all mutants exhibited a phase III with rates similar to that of wild type (Table 1). Observing a similar phase III for the ESCRT-defective variant appears counterintuitive since this variant is known to exhibit a defect in particle release. However, a quantitative analysis of the mobility of these particles showed differences in the mobility as compared to the wt measurements. The averaged diffusion coefficient determined from 170 trajectories was *D* = (1.5±1.9)·10^−4^ µm^2^/s. In three instances, rapid movement on the order of ∼10^−3^ µm^2^/s was observed, but these punctae resided at the cell edge and displayed collective motion indicating that the particles had not been released. A change in fluorescence intensity in TIRFM without concomitant change in instantaneous velocity can be caused by an altered axial position of a late budding structure. Due to the exponential decay of the evanescent wave in TIRF mode, virons or late budding structures that are close to the coverslip would lead to an increase in the fluorescence intensity of the assembly site observed in TIRFM but not in WF. We exploited the sensitivity of TIRFM to the axial position of the fluorescent protein cluster by comparing the distributions in fluorescence intensity of the TIRF and WF channels. A time course of the TIRF (Figure S7B) and WF (Figure S7C) fluorescence intensity distributions at individual assembly sites is shown in Figure S7 for three different time windows (0–15 min, 15–30 min, 30–45 min after their initial detection). The histogram of fluorescence intensities determined by TIRF microscopy revealed three discernible peaks indicating different brightness classes, one centered about 500 counts, a brighter subpopulation at ∼1,000 counts, increasing in amplitude with time, and a third subpopulation observed as a small shoulder around 2,000 counts. Figure S7A shows an example image indicating individual particles from the different brightness classes. The brighter subpopulations were hardly discernible in the WF histograms (compare Figure S7B, D with C, E, respectively), indicating that they represent protruding Gag clusters, i.e. trapped virions or arrested budding structures close to the cover slip. The late domain deficient HIV variant exhibited a second bright subpopulation in TIRF mode, which was detectable as a shoulder in the brightness histogram (Figure S7D, E) and is more prevalent at the later time points. In this case, the bright fraction may represent arrested late budding structures.

### Origin of Gag molecules recruited to the budding site

**Figure 5:**
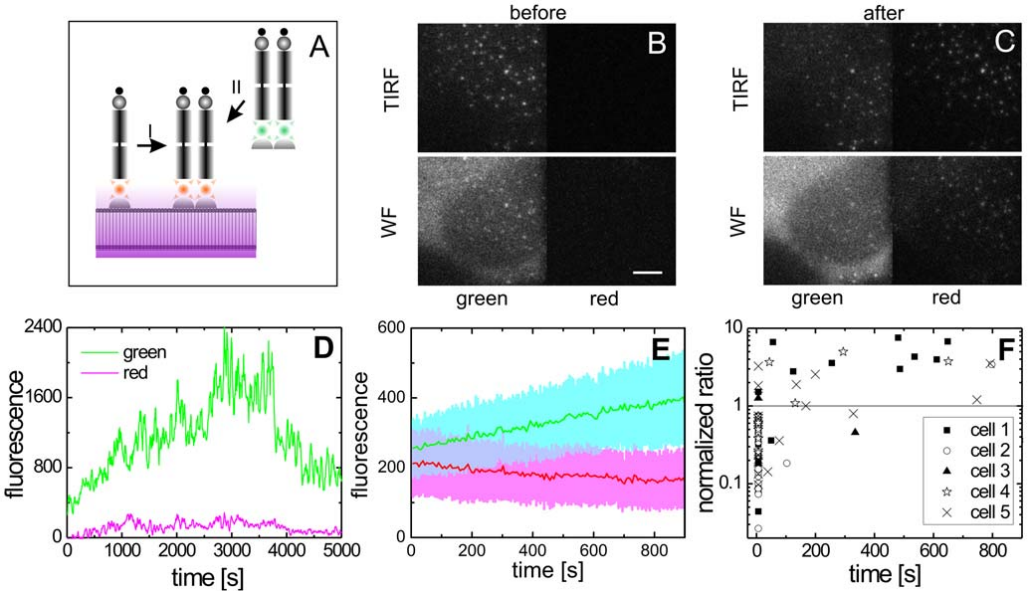
Recruitment of Gag to the budding site. (A) Scheme of the photoconversion experiment designed to determine the source of Gag molecules recruited to an assembly site. Recruitment can either occur from the surrounding plasma membrane (I) or directly from the cytosol (II). By calculating the ratio of fluorescence intensities in green and red channels at the assembly site with respect to the same ratio calculated from the local background, the origin of the recruited Gag molecules could be determined. (B–C) Live-cell images of a HeLa cell transfected with a mixture of pCHIV andpCHIV^mEosFP^ before photoconversion (B) and directly after photoconversion (C). The scale bar signifies 5 µm. (D) Intensity trajectory from an individual Gag cluster that appeared 26 s after photoconversion. (E) The average fluorescence intensity with 488 nm excitation (green line) and with 561 nm excitation (red line) of the plasma membrane measured in the vicinity of 23 assembly sites that were detectable before photoconversion as a function of time after photoconversion. The standard deviation of the individual traces is given as a halo. (F) The ratio of fluorescence intensity after green excitation to the fluorescence signal after red excitation normalized to the same ratio determined from the local background measured for 102 different assembly sites in five cells.

In Phase I, individual Gag clusters form rapidly at the plasma membrane and further Gag molecules are recruited to the growing assembly sites on a time scale of minutes. To determine whether Gag molecules arrive at the assembly site directly from the cytosol or by lateral diffusion within the plasma membrane, we made use of an HIV-1 variant labeled with a thermostable mutant of the photoconvertible protein mEosFP (kindly provided by J. Wiedenmann). This protein can undergo irreversible photoconversion from green to red emission upon exposure to 405-nm irradiation [42],[43]. Photoconversion using TIRF excitation results in preferential conversion of Gag.mEosFP molecules in the plasma membrane whereas cytosolic Gag will be mostly unaffected (Figure 5A). Ideally, when Gag is recruited exclusively from the cytosol, the green fluorescence intensity should increase whereas the red fluorescence intensity remains constant. In contrast, recruitment of Gag from the surrounding membrane would result in signal increase in the red channel, whereas the intensity in the green channel would remain constant.

Figure 5 shows TIRF (upper panels) and WF (lower panels) views of a cell producing HIV-1^mEos^ before (Figure 5B) and after (Figure 5C) photoconversion using TIRF mode. Arrival of new Gag molecules at the sites of virus assembly was monitored simultaneously in the green and red channels. In the representative experiment shown here, approximately 30% of mEosFP was photoconverted upon illumination for 90s with 405 nm light. Illumination in TIRF mode converted primarily Gag.mEosFP molecules at the plasma membrane and very little increase in cytosolic background was observed in the red channel (Figure 5C, *lower right panel*). As non-photoconverted Gag.mEosFP is continually recruited to the membrane, we focused our analysis on assembly sites that appeared within 15 min after photoconversion. Figure 5D shows the fluorescence intensity from a single assembly site that was first observable 26 s after photoconversion. The increase in the fluorescence intensity of the green channel was much more pronounced than that of the red channel.

The amount of non-photoconverted Gag.mEosFP to photoconverted Gag.mEosFP in the plasma membrane is continually changing during the experiment. This is due to, for example, diffusion of the labeled Gag within the plasma membrane, new non-photoconverted Gag being delivered to the plasma membrane, late maturation of the chromophore or the different photobleaching rates for the photoconverted and non-photoconverted species. Therefore, we monitored the fluorescence intensity of the plasma membrane (Figure 5E). The fluorescence intensity after 488 nm excitation (i.e. the non-photoconverted species) increased with time whereas the fluorescence intensity after 561 nm excitation (i.e. the photoconverted species) was gradually decreasing. To account for the time-dependent changes in the fluorescent signal of the non-photoconverted and photoconverted species in the plasma membrane, we define a normalized ratio, 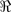, which is the ratio of the signal after 488 nm excitation to the signal after 561 nm excitation divided by the same ratio calculated for the background in the neighborhood of the respective assembly site at the same time point. This normalization accounts for any differences due to incomplete photoconversion, slow maturation of the mEosFP chromophore, changes in the concentration of the non-photonconverted and photoconverted Gag.mEosFP due to diffusion within the membrane, delivery of new Gag molecules to the plasma membrane, photobleaching, or potential Förster Resonance Energy Transfer between the non-photoconverted and photoconverted species. In addition, the background used for the analysis is in the near vicinity of the assembly site meaning that we are not sensitive to undulations of the plasma membrane on the lateral scale of ∼1.5 µm or larger. However, preexisting budding structures would have a higher percentage of photoconversion due to the closer proximity of the virion to the cover slip than the local background. Accordingly, this can be observed for the assembly sites that were present before photoconversion, plotted at *t* = 0 in Figure 5F, which show a distribution of normalized ratios with an average value significantly lower than one. When assembly sites recruit membrane-bound Gag molecules, the ratio of fluorescence intensities from the individual assembly site will be approximately equal to the background signal coming from the plasma membrane. Technically, the background contains a contribution of fluorescence from cytosolic non-photoconverted Gag.mEosFP that is still observable in the TIRF evanescent field. This signal does not appear in the fluorescence intensities of the individual assembly sites as they are background corrected. Therefore, the ratio of non-photoconverted signal to photoconverted signal will be lower than that calculated for the local background: 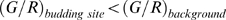 when Gag is accumulated from the plasma membrane. In the opposite case, when the majority of Gag is recruited from the cytosol, this ratio in the assembly site would be greater than that of the local background. Therefore, we plotted the normalized ratio, 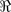, and compared it to unity (Figure 5F).

For the newly generated assembly sites, we determined this normalized ratio either at the onset of assembly or in the time window of 3–5 minutes after assembly has begun. Thus, we could also investigate whether Gag is recruited from different locations during assembly. 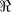 at the onset of nucleation was determined by extrapolating linear fits of the early points of the fluorescence signal and the background to the time of first detection. The normalized ratios are plotted in Figure 5F as a function of time of appearance after photoconversion. At early time points after photoconversion, we observed some variability and the newly formed assembly sites had normalized ratios both greater than and less than one. However, after ∼100 s, almost all new assembly sites exhibited normalized ratios above one. This observation suggests that nucleation of assembly sites involves Gag molecules coming directly from the cytosol or Gag that has only recently been delivered to the plasma membrane. As we cannot distinguish between Gag molecules that were delivered directly to the assembly site from Gag molecules that were delivered to the plasma membrane in the direct vicinity of the assembly site and immediately incorporated into the assembly site, it is also possible that Gag molecules may first bind briefly to the plasma membrane (∼100s) before being incorporated into the assembly site. Regardless of whether the Gag is delivered directly from the cytosol to the assembly site or interacts briefly with the plasma membrane before incorporation into the assembly site, the Gag is *effectively* being delivered from the cytosol. By analyzing the normalized ratio in the time interval of 3 to 5 minutes after initiation of the individual sites, we determined that the assembly sites grow with the majority of Gag coming *effectively* from the cytosol (Figure S8).

**Figure 6:**
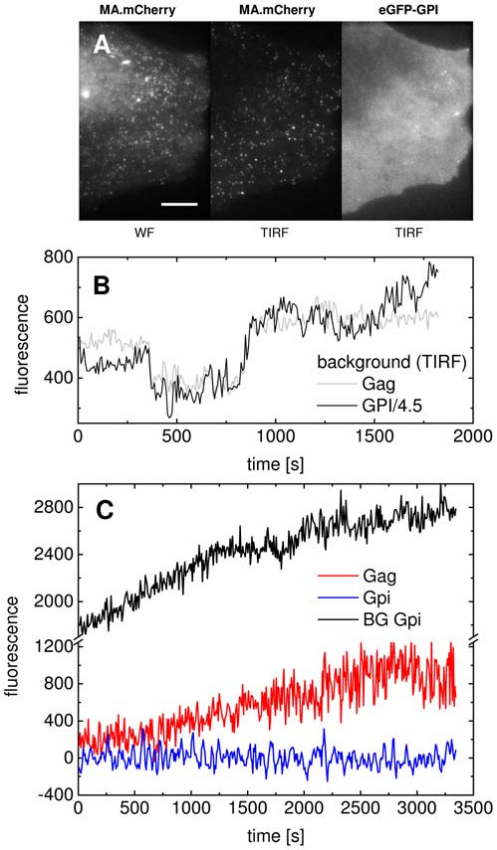
Comparison of the dynamics of eGFP-GPI and HIV-1 Gag during viral assembly. (A) Live-cell images of mCherry-labeled Gag in wide field (*left panel*) and TIRF (*middle panel*) as well as eGFP-GPI imaged in TIRF (*right panel*); the scale bar represents 10 µm. (B) The fluorescence intensity of eGFP-GPI observed in the green channel at the location of the tracked HIV^mCherry^ assembly site (black line) and the background signal in the red channel derived from a region surrounding the tracked particle (grey line). The fluorescence intensity from the eGFP channel is divided by a factor of 4.5. Fluctuations of the plasma membrane in the TIRF evanescent field were detectable in both traces, validating usage of the Gag background signal as an indicator of membrane fluctuations in our analyses. (C) The background GPI signal (grey) increased over time indicating the delivery of new eGFP-GPI to the membrane. No enhancement of the eGFP-GPI signal (blue) was found at the individual HIV budding site, whereas Gag (red) increased.

Within the context of current controversial theories of HIV-1 assembly (reviewed in [15]), our results support the model that virion formation occurs exclusively at the plasma membrane, at least in HeLa cells. We observed no evidence for Gag containing vesicles arriving at the plasma membrane. As a control measurement, we performed dual-color experiments using eGFP-GPI [44] as a membrane marker along with Gag.mCherry (Figure 6). Interestingly, no difference in eGFP-GPI concentration was seen at assembly sites compared to the surrounding background (Figure 6C). This suggests that there is no preferred incorporation or exclusion of eGFP-GPI from the budding site. Similar results were obtained using a myristoylated and palmitylated version of eYFP (Figure S9 [45]). Thus, while HIV-1 particles display a ‘raft’-like lipid composition [46], the budding sites likely represent a specialized form of DRM distinct from other ‘raft’-like membrane domains. Bright bursts of GFP fluorescence in eGFP-GPI expressing cells as observed in supporting Video S6 most likely originate from fusion of GPI-loaded vesicles with the plasma membrane. Such bursts were not detected in measurements with Gag.eGFP. While this does not rule out that small numbers of Gag molecules are delivered to the plasma membrane via vesicles, the data suggest that the delivery of larger assemblies of Gag molecules through a vesicular compartment is not involved in the Gag assembly process. We also did not detect pre-assembled large Gag multimers being transported to the plasma membrane in our SDCM experiments. However, our results suggest that HIV-1 assembly is nucleated by Gag oligo- or multimers at the plasma membrane which are subsequently extended by further Gag molecules recruited effectively from the cytosol with perhaps a small contribution from Gag molecules diffusing laterally in the plasma membrane.

### Conclusions

In summary, our results indicate that formation of HIV-1 assembly sites occurs during a relatively short period after onset of assembly in an individual cell, while particle production is completely asynchronous in the overall culture. Virus release occurs from individual assembly sites and not from preformed budding platforms. Assembly of the Gag shell (phase I) occurred with a characteristic time of ∼200 s (or ∼8–9 min for 90% assembly), slightly slower than the 5–6 min recently reported by Jouvenet et al. [22]. Using the instantaneous velocity of the particle as a marker for extracellular release, we provide a first estimate for the duration from appearance of an assembly site to particle release of a complete HIV-1 particle from HeLa cells: approximately 25 minutes. Assembly of the Gag shell appears to constitute only a minor part of this period indicating that traversing the membrane and fission are the rate-limiting stages in virion formation. Similar results were obtained for release from the ventral membrane measured via TIRF microscopy and from the dorsal membrane determined using SDCM. Using a photoconvertable label, we established that the Gag molecules that participate in nucleation of a new assembly site and in bud growth are recruited preferentially from the cytosolic pool of Gag molecules and from recently membrane-attached Gag. We found that, in spite of DRM-like composition of the lipid envelope, GPI was not enriched at the budding site. The described results add essential dynamic information to our picture of virus release and provide an experimental basis for interfering with this stage of virus replication.

## Materials and Methods

### Plasmids

Construction of plasmids pCHIV, pCHIV.eGFP, pCHIV.mCherry, their late-domain defective variants, which contain a PTAP to LIRL exchange in the p6 domain, and of the protease-negative variants has been described elsewhere [24],[25]. Variants defective in Vpu were cloned by exchange of an EcoRI-XhoI fragment from plasmid HIV-1 NL4-3 p210-13 3′ D-Vpu [47]. pCHIV.mEOS was constructed the same way, based on a construct encoding folding-optimized monomeric EosFP in the pQE32 vector kindly provided by J. Wiedenmann [42],[43].

### Cell culture and preparation of labeled particles

HeLa and 293T cells were maintained in DMEM supplemented with 10% FCS. For microscopic analyses, HeLa cells were transfected using FuGene6 (Roche) according to the manufacturer's instructions and imaged in phosphate buffered saline supplemented with 0.9 mM CaCl_2_ and 0.5 mM MgCl_2_. As a standard for the fluorescence intensity of complete viruses, particles were purified by ultracentrifugation from the supernatant of 293T cells transfected by CaPO_4_ precipitation with a mixture of pCHIV and pCHIV.eGFP in a molar ratio of 1∶1 as previously described [25]. The particle concentration was measured by p24 ELISA.

### Single virus tracing microscope

A microscope capable of synchronous switching between TIRF- and WF-microscopy on a frame by frame basis was built for this study. A scheme of the experimental setup is shown in Figure S1. The excitation paths were combined using a polarizing beam splitter so that all wavelengths could be used for each type of microscopy. Mechanical shutters were used to switch between microscopy modes. In this configuration, photoconversion could also be performed in the TIRF mode, allowing photoconversion of plasma membrane associated Gag.mEOS molecules. The detection path was split into two channels and imaged on different portions of an EMCCD camera (Andor Technology). For comparison, experiments were also performed on a SDCM. A more detailed description of the setups is given in the Supporting Text S1.

### Particle tracking algorithm

Single virus tracing was applied to track individual Gag clusters over the time course of assembly. The tracking routine consisted of four steps: (i) particle identification, (ii) prediction of the particle position using a spatial-temporal filter, (iii) global mapping of detected particles to the predicted particle position and (iv) final assignment of particles within the movie based on the estimates of the predicted and detected particle position. For dual channel tracking, an additional step was incorporated to take the union of trajectories found in each channel separately. In addition to the position of the tracked particle, the total intensity at the particle position was determined along with the background about the particle, which was then subtracted from the total intensity to provide the particle intensity. A detailed description of the tracking algorithm can be found in the Supporting Text S1 and in [48].

## Supporting Information

Text S1 Supporting Methods
(0.10 MB PDF)

Figure S1 Schematic diagram of the experimental setup. The excitation wavelength is selected with an acousto-optic tunable filter and can be alternated frame by frame. A polarizing beamsplitter is used to separate and combine the TIRF excitation path and WF excitation path. The shutters were synchronized with the camera such that alternating excitation methods could be used with a time resolution down to 30 ms/frame.
(0.22 MB JPG)

Figure S2 The time course of the appearance of assembly sites. The number of detected assembly sites on a single cell is plotted as a function of time for a representative measurement. Starting from the time point where the first clusters of Gag.eGFP were detected (t = 0), a large increase in the number of assembly sites was typically seen within one hour.
(0.26 MB JPG)

Figure S3 Types of assembly kinetics. Three types of fluorescence increase were observed during virus assembly. The vast majority of traces (∼80%) followed a saturating exponential behavior (A); in 10% to 20% of the clusters, a linear growth pattern was observed (B); occasionally (<3%) we observed traces that displayed an exponential growth until a plateau was reached (C).
(0.29 MB JPG)

Figure S4 Assembly monitoring using SDCM. To ensure that the rates determined from TIRFM were not influenced by the strong dependence of the intensity of the distant of the fluorophores from the cover slip, additional experiments were performed using SCDM. The results of Phase I for HIV^eGFP^ are shown for an individual assembly site (A) and for an average from 60 traces (B). The averaged value is shown in black along with the standard deviation of the data in grey and an exponential fit to the data in red.
(0.33 MB JPG)

Figure S5 Histogram of the duration of phase I and II for HIV wild-type (A, B) and HIV ΔVpu (B,C) assembly. The time from the first appearance of the assembly site until the onset of phase III was determined for 160 (wt) and 69 (ΔVpu) individual assembly sites respectively (A, C). The duration of phase II was estimated as the time from 90% completion of phase I until the onset of phase III and is plotted in panels B and D for wt and ΔVpu cells respectively. A binning of 200 s was used for all of the histograms.
(2.48 MB JPG)

Figure S6 Release of HIV-1 observed using SDCM. (A) An image from Video S6 showing the assembly and release of an HI virion monitored on the dorsal membrane of the cell using SDCM (A). The trajectory, color coded to show the three phases, assembly phase (phase I, red), plateau region (phase II, yellow), and release (phase III, cyan) is overlaid. The beginning of the trajectory is marked with an arrowhead, while the arrow is pointing to a particle being followed. Scale bar = 1 µm. The fluorescence intensity (B) and instantaneous velocity (C) are shown for the above trajectory with the same color coding.
(0.19 MB JPG)

Figure S7 Fluorescence Intensity Distribution of Individual Assembly Sites. Image of a HeLa cell transfected with pCHIV/pCHIV^eGFP^ imaged in TIRF mode (A); colored arrows indicate individual punctae that belong to different fluorescence intensity classes displayed in panel (B). Histograms of fluorescence intensities normalized per frame (B) for HIV/HIV^eGFP^ in TIRF mode, (C) HIV/HIV^eGFP^ in WF mode, (D) HIV(late-)/HIV^eYFP^(late-) in TIRF mode and (E) HIV(late-)/HIV^eYFP^(late-) in WF mode. Histograms correspond to 0–15 (*left panels*), 15–30 min (*middle panels*) and 30–45 min (*right panels*) after the start of data collection. As measurements were performed with different camera settings, the fluorescence intensities have been normalized to allow a direct comparison between experiments.
(1.01 MB JPG)

Figure S8 Recruitment of Gag after nucleation. The ratio of fluorescence intensity after green excitation to the fluorescence signal after red excitation normalized to the same ratio determined from the local background. Values are averaged over the time interval of 3 to 5 minutes after nucleation of the budding site. The normalized ratio is still greater than 1, indicating that the majority of Gag being incorporated into the budding at this time is recruited from the cytosol.
(0.29 MB JPG)

Figure S9 Comparison of the dynamics of MyrPalm-mYFP and HIV-1 Gag during viral assembly. (A) Images of mCherry-labeled Gag in wide field (*left panel*) and TIRF (*middle panel*) and MyrPalm-eYFP in TIRF (*right panel*) obtained at 30 hpt; the scale bar represents 10 µm (B) Fluorescence intensities of Gag.mCherry and MyrPalm-YFP as a function of time for an individual budding site. No concomitant accumulation of the MyrPalm.mYFP signal (shown in blue) with the Gag signal (show in red) was observed.
(0.62 MB JPG)

Video S1 Alternating TIRFM and WF measurements of HIV assembly and budding in a HeLa cell transfected with pCHIV and pCHIV^eGFP^. A single frame from this movie is shown in Figure 1. Data were collected at a rate of 0.6 frames/s for a total duration of 1∶57 hr∶min.
(9.50 MB AVI)

Video S2 A potential platform budding event from a cell transfected with pCHIV and pCHIV^eGFP^. The apparently subsequent release of three particles from a single diffraction-limited region are shown. Data were collected at a rate of 0.16 frames/s. The duration of the movie is 30 min.
(0.87 MB AVI)

Video S3 A HeLa cell producing HIV.eGFP particles imaged using a spinning-disk confocal microscope. A maximum intensity projection over 10 z-slices with a spacing 0.3 µm between slices (or a total of 3 µm) successfully eliminated the intensity fluctuations caused by the z displacement, guaranteeing that the measured kinetics are not influenced by axial fluctuations of the particle in the TIRFM evanescent field. The total movie duration is 70 min.
(7.48 MB AVI)

Video S4 This movie shows the budding of an individual HIV-1 virion starting from nucleation and assembly through to release. The trajectory is color coded to show the three phases, phase I (assembly) in red, phase II (plateau region) in yellow and phase III (release in this case) in light blue. The movie was collected at 0.6 frames/s. The total movie duration is 45 min.
(3.67 MB AVI)

Video S5 The budding of a single virion from nucleation and assembly to release measured using SDCM. The trajectory is color coded to show the three phases, phase I (assembly) in red, phase II (plateau region) in yellow and phase III (release in this case) in light blue. The movie was collected at 0.06 frames/s. The total movie duration is 55 min.
(1.66 MB AVI)

Video S6 The assembly of Gag clusters was investigated using HIV^mCherry^ while the membrane was labeled with eGFP-GPI as a marker for investigating fluctuations of the plasma membrane. Alternating frames were collected in TIRFM and WF mode. A frame from this movie is shown in Figure 6. The duration of the movie is 56 min.
(2.83 MB AVI)
