# Supplementary material for: Dynamics of HIV-1 Assembly and Release
Source: PLoS Pathog. 2009 Nov 6;5(11):e1000652. doi: 10.1371/journal.ppat.1000652 (PMC2766258; doi:10.1371/journal.ppat.1000652)
Supplement: Text S1 — Supporting Methods (0.10 MB PDF) [file ppat.1000652.s001.pdf]

SUPPORTING INFORMATION

Table of contents **Page**

Table of contents	Page
Supporting Material and Methods	2-6
Supporting References	7

SUPPORTING MATERIAL AND METHODS

Single Virus Tracing Microscope

Image sequences were acquired on a home-built combined wide-field (WF)/total internal reflection fluorescence (TIRF) microscope with fast switching capability between the two modes (a schematic diagram is shown in Figure S1). We used a 490-nm frequency-doubled diode laser (Picarro, Newport, Mountain View, CA) to excite EGFP, EYFP and the green form of mEosFP. A 561-nm diode-pumped solid state laser (CrystaLaser, Reno, NV) was used to excite the red form of mEosFP as well as mCherry. For photoconversion of mEosFP, we used a 405-nm diode laser (Coherent, Santa Clara, CA). Excitation wavelength selection was performed by an acousto-optic tunable filter (AA sa, Orsay Cedex, France) coupled into a 100-μm multimode fiber (AMS Technologies, Munich, Germany). The fiber was linked to a shaker in order to provide homogeneous illumination of the sample. The output of the fiber was split in two optical paths by a polarizing beam-splitter (Newport Corporation, Irvine, CA) and these paths were later combined on another polarizing beam-splitter at the back port of a Nikon TE300 microscope (Nikon Corporation, Tokyo, Japan). After the beam was fed into the microscope, the excitation beam was reflected by a z488/568rdc dichroic beamsplitter (Chroma, Rockingham, VT) into the objective.

In the wide-field path, the fiber output was projected onto an aperture that was placed in a conjugate plane of the 60x N.A. 1.49 Apo TIRF oil immersion lens (Nikon) and defined the sample illuminated area. Sample illumination using total internal reflection was performed by focusing the laser beam onto the edge of the back focal plane of the same objective such that the transmitted light from the objective was incident at angles larger than the critical angle for the coverslip/buffer interface. We used a telescope to expand the excitation beam before focusing it on the back focal plane of the objective to increase the field-of-view illuminated by TIRF excitation. The telescope was also used to adjust the beam divergence at the exit of the objective, since objective-type TIRF requires low divergence of the excitation beam. Switching between the two excitation modes was performed by two mechanical shutters Uniblitz VS14S2S0 (Vincent Associates, Rochester, NY).

Fluorescence was collected by the same objective and passed through the excitation dichroic mirror followed by a dual-band GFP/dsRed emission filter (Chroma). The green and red spectral channels were separated using a Q565LP dichroic mirror (Chroma). Both channels were projected onto two separate areas of a EMCCD camera (DU-897 Ixon+, Andor, Belfast, Ireland). Shutter and AOTF control was performed by an PCIM-DDA06/16 card (Measurement Computing Corporation, Norton, MA) and Andor IQ 1.6 software (Andor).
A spinning-disk confocal microscope (SDCM) was purchased from Andor (Revolution System). Briefly, the microscope is based on a Nikon TE2000-E, which is equipped with 100x N.A.1.49 Apo TIRF oil immersion lens (Nikon) and CSU-10 scanning head (Yokogawa Electric Corporation, Tokyo, Japan). A combination of 488-nm laser excitation, FF01-525/50-25 emission filter (Semrock, Rochester, NY) and EMCCD camera (DU-897 Ixon+, Andor) was used to observe the individual Gag.eGFP clusters.

Particle Tracking Algorithm

As assembly sites were found to be mobile structures (see Supporting Movies), tracking of individual particles was necessary to analyze the changes in fluorescence intensity over time. A detailed description of the tracking software has been reported elsewhere [1]. Briefly, we have developed a probabilistic approach for automated tracking of multiple virus particles [2]. Each virus particle is represented by a 2D Gaussian function, which is parameterized by the position of the virus particle, the peak intensity, and the standard deviation σ_{xy}. For each time point of an image sequence, our tracking approach comprises four steps: (i) particles corresponding to virus particles are detected using a Laplacian-of-Gaussian filter, also known as a spot-enhancing filter [3,4], with a standard deviation of $\sigma = 1.5$ pixels and a dynamically adjusted threshold to account for the increasing background signal with time. The threshold is computed from the background intensity level plus a factor c times the standard deviation of the background noise. To compensate for the changing background fluorescence, the factor c was adjusted linearly with time. To identify virus particles, we employed a connected-components labeling algorithm (8-connectivity), which groups spatially adjacent detected pixels by analyzing the local region (consisting of eight pixels) around each considered pixel. The position of

each particle is determined by computing the intensity-weighted center-of-mass. (ii) a prediction for the position of each tracked particle in the current image is determined using a spatial-temporal filter (in this case, we used a Kalman filter with a Brownian motion model). (iii) each predicted particle is matched to a detected particle in the current image via a global nearest-neighbor approach [5]. (iv) based on the predicted and detected position estimates, the final position estimate of a tracked particle is computed using the spatial-temporal filter.

Tracking was often performed simultaneously in two channels. This could be either red and green detection or, alternatively, TIRF and WF images. In the latter case, particles were detected in the TIRF channel where the signal-to-background ratio is the highest and the fluorescence intensity calculated from the identical position in the WF channel. For dual-color tracking, we added an additional detection step to our previous approach. We first applied the detection algorithm as described above (based on the Laplacian-of-Gaussian filter) to each channel separately. This results in two sets of detections, one for each channel. A consolidated set of detections (which is used for tracking) is determined by applying a union operation to the two sets. If a virus particle is detected in both images, then only the detection from one channel is included in the consolidated set. For deciding whether this is the case, we use a nearest-neighbor approach, where the maximum distance between two corresponding detections is set to 2 pixels (in some cases we used a distance of 1 pixel).

For computing the necessary image intensity statistics, we employ the original image data. The average intensity of a particle is determined over a circular region with a radius of $\lceil \sigma_{xy} \rceil$ centered at the estimated position of the particle (where $\lceil x \rceil$ denotes the smallest integer value of x not less than x). The parameter σ_{xy} is determined by computing the width of a bounding box enclosing the detected and identified virus particles. The average intensity of the local background for each particle is computed over an annulus defined by two circles: the inner circle has a radius of $\lceil \sigma_{xy} \rceil$ and the outer circle a radius of $\lceil 5\ \sigma_{xy} \rceil$; both circles are centered at the computed position of the particle. To determine the signal intensity of a particle, we subtract the average intensity of the local background from the average intensity of the particle.

Background Correction

From the analysis routine, both the background corrected signal intensity and the background signal were determined. Local background correction was used and monitored to ensure that the changes in fluorescence signal observed were not correlated to changes in the background intensity. To test whether the background provided accurate information regarding fluctuations of the plasma membrane, experiments were also performed with GPI anchored eGFP as a membrane marker together with $HIV^{mCherry}$ (Figure 6 and Video S6). The extent of membrane fluctuations observed varied from cell to cell. Figure 6 depicts the correlation between membrane motions observed using either eGFP.GPI or the local background, showing that both methods yielded equivalent results; comparison with local background was used in all further measurements. The local background results for the clusters tracked in Fig. 1C are shown for both TIRF and WF data. The lower background signal from TIRF resulting in the better signal-to-noise ratio is evident.

Data Analysis

To extract the overall dynamics of the assembly process, a cross-correlation analysis to synchronize and average multiple traces was used. In general, all individual intensity trajectories exhibited three main phases, Phase I (an increase in fluorescence intensity), Phase III (a subsequent decrease in fluorescence intensity), and a plateau region of variable duration separating these phases (Phase II). As the assembly of Gag clusters occurs asynchronously, the traces had to be synchronized before being averaged to determine the overall form.

The initial phase was easily synchronized by using the time point at which the particle was initially recognized by the software. To discriminate budding from sporadic detection of complete particles, traces were selected that displayed signals for longer than 300 frames. In addition, the average intensity slope of the first 300 frames (~500 sec) was required to be positive, both in the TIRF and wide-field channels. Synchronization and averaging of Phase I yielded an increase in fluorescence that can be best approximated by the saturating exponential:

$$y = A_I\left(1-\exp\left[-k_I\left(t-t_0\right)\right]\right) \tag{S1}$$

As the time point of detection is susceptible to errors, these trajectories were synchronized a second time using a cross-correlation approach. The individual intensity trajectories were cross-correlated with a template function, the saturating exponential for Phase I. From the maximum of the cross-correlation function, the shift providing the best overlap with the template function was determined. In addition, traces that did not correlate well with the template function were removed by requiring a minimum cross-correlation amplitude. The traces were also manually inspected and outliers were removed when necessary. The trajectories were then averaged in the overlap region and the standard deviation was calculated. The averages were fit to a saturating exponential function using a nonlinear least-square routine in order to calculate the rate constants. The rates determined did not depend on the criterion used to select the traces for averaging.

For determination of the dynamics of Phase III, a linear function with a negative slope was used as the template function for synchronization. The result of the cross-correlation analysis did not depend on the slope of the template function. The corresponding averaged signal was fitted to the decaying exponential with an offset:

$$y = A_{III}\exp\left(-k_{III}\left(t-t_0\right)\right)+B_{III}\,. \tag{S2}$$

The mean rates from the different cells were then averaged together where the rate from each cell was weighted by the number of trajectories for that cell. The averaged fluorescence intensity traces and fits are shown in Fig. 3 and given in Table 1.

SUPPORTING REFERENCES

1. Godinez WJ, Lampe M, Worz S, Muller B, Eils R, et al. (2009) Deterministic and probabilistic approaches for tracking virus particles in time-lapse fluorescence microscopy image sequences. Med Image Anal.
2. Godinez WJ, Lampe M, Worz S, Muller B, Eils R, et al. Tracking of Virus Particles in Time-Lapse Fluorescence Microscopy Image Sequences; 2007. pp. 256-259.
3. Marr D, Hildreth E (1980) Theory of edge detection. Proc R Soc Lond B Biol Sci 207: 187-217.
4. Sage D, Neumann FR, Hediger F, Gasser SM, Unser M (2005) Automatic tracking of individual fluorescence particles: application to the study of chromosome dynamics. IEEE Trans Image Process 14: 1372-1383.
5. Sbalzarini IF, Koumoutsakos P (2005) Feature point tracking and trajectory analysis for video imaging in cell biology. J Struct Biol 151: 182-195.
