# Supplementary figures and images for: Dynamics of HIV-1 Assembly and Release
Source: PLoS Pathog. 2009 Nov 6;5(11):e1000652. doi: 10.1371/journal.ppat.1000652 (PMC2766258; doi:10.1371/journal.ppat.1000652)

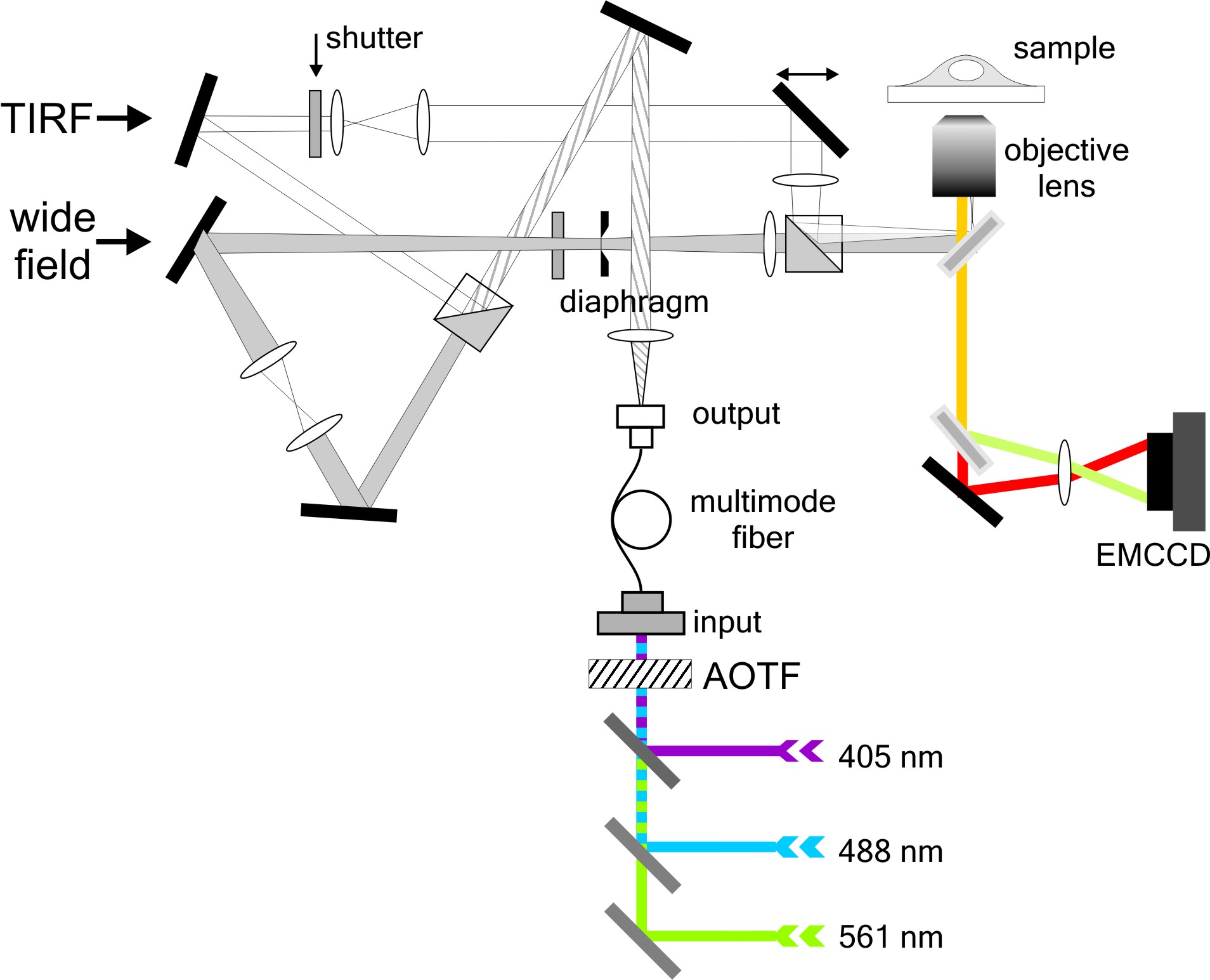

Supplement: Figure S1 — Schematic diagram of the experimental setup. The excitation wavelength is selected with an acousto-optic tunable filter and can be alternated frame by frame. A polarizing beamsplitter is used to separate and combine the TIRF excitation path and WF excitation path. The shutters were synchronized with the camera such that alternating excitation methods could be used with a time resolution down to 30 ms/frame. (0.22 MB JPG) [file ppat.1000652.s002.jpg]

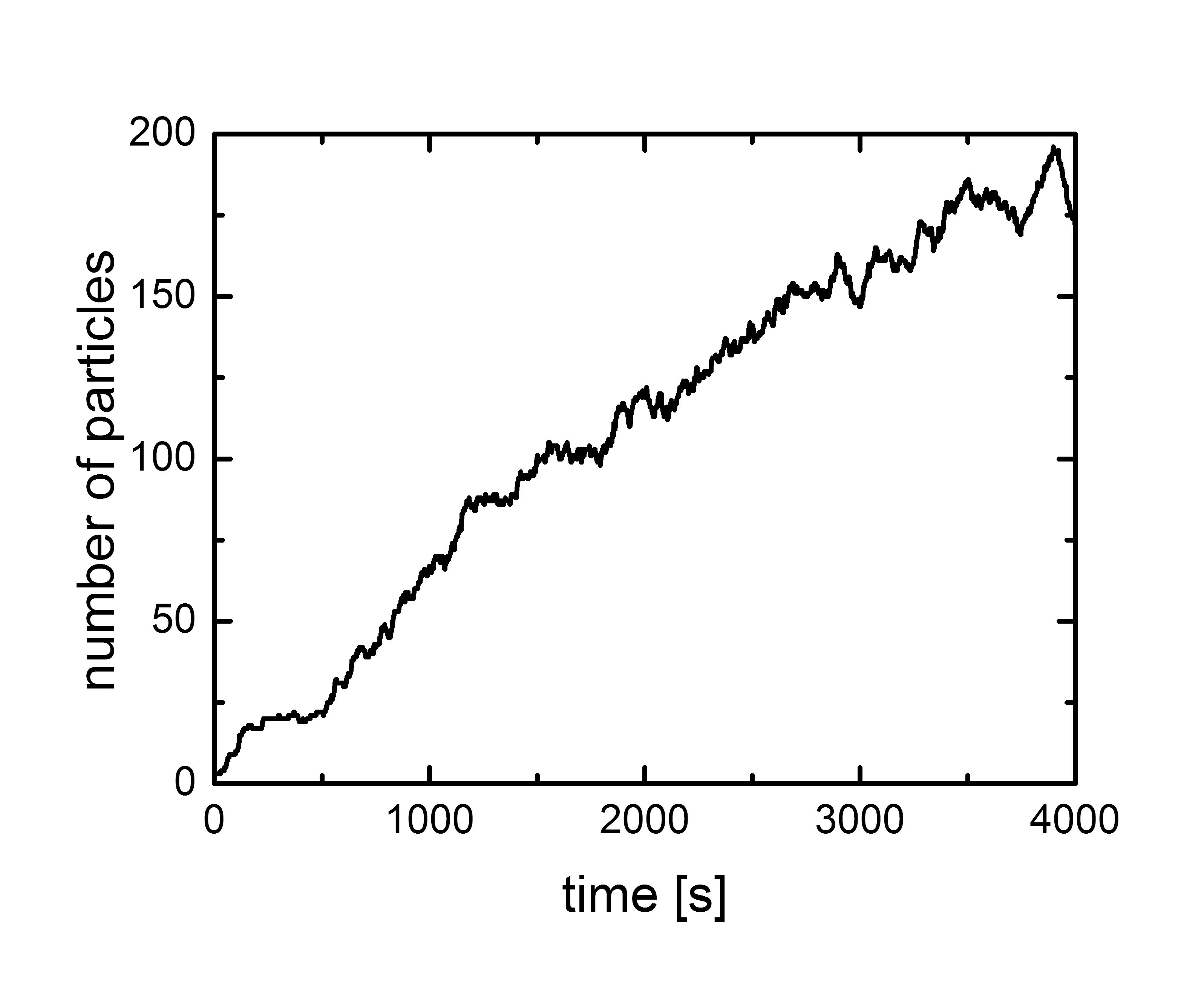

Supplement: Figure S2 — The time course of the appearance of assembly sites. The number of detected assembly sites on a single cell is plotted as a function of time for a representative measurement. Starting from the time point where the first clusters of Gag.eGFP were detected (t = 0), a large increase in the number of assembly sites was typically seen within one hour. (0.26 MB JPG) [file ppat.1000652.s003.jpg]

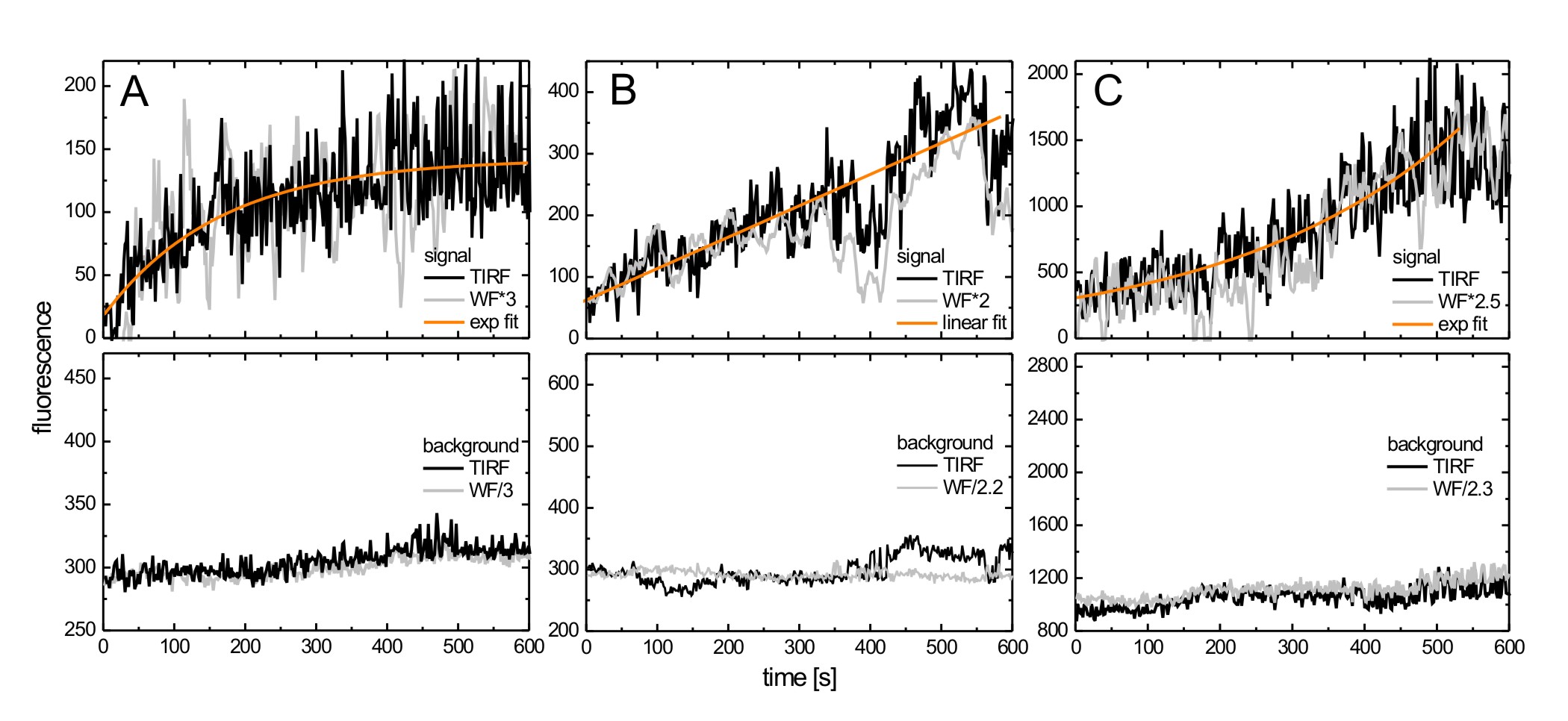

Supplement: Figure S3 — Types of assembly kinetics. Three types of fluorescence increase were observed during virus assembly. The vast majority of traces (∼80%) followed a saturating exponential behavior (A); in 10% to 20% of the clusters, a linear growth pattern was observed (B); occasionally (<3%) we observed traces that displayed an exponential growth until a plateau was reached (C). (0.29 MB JPG) [file ppat.1000652.s004.jpg]

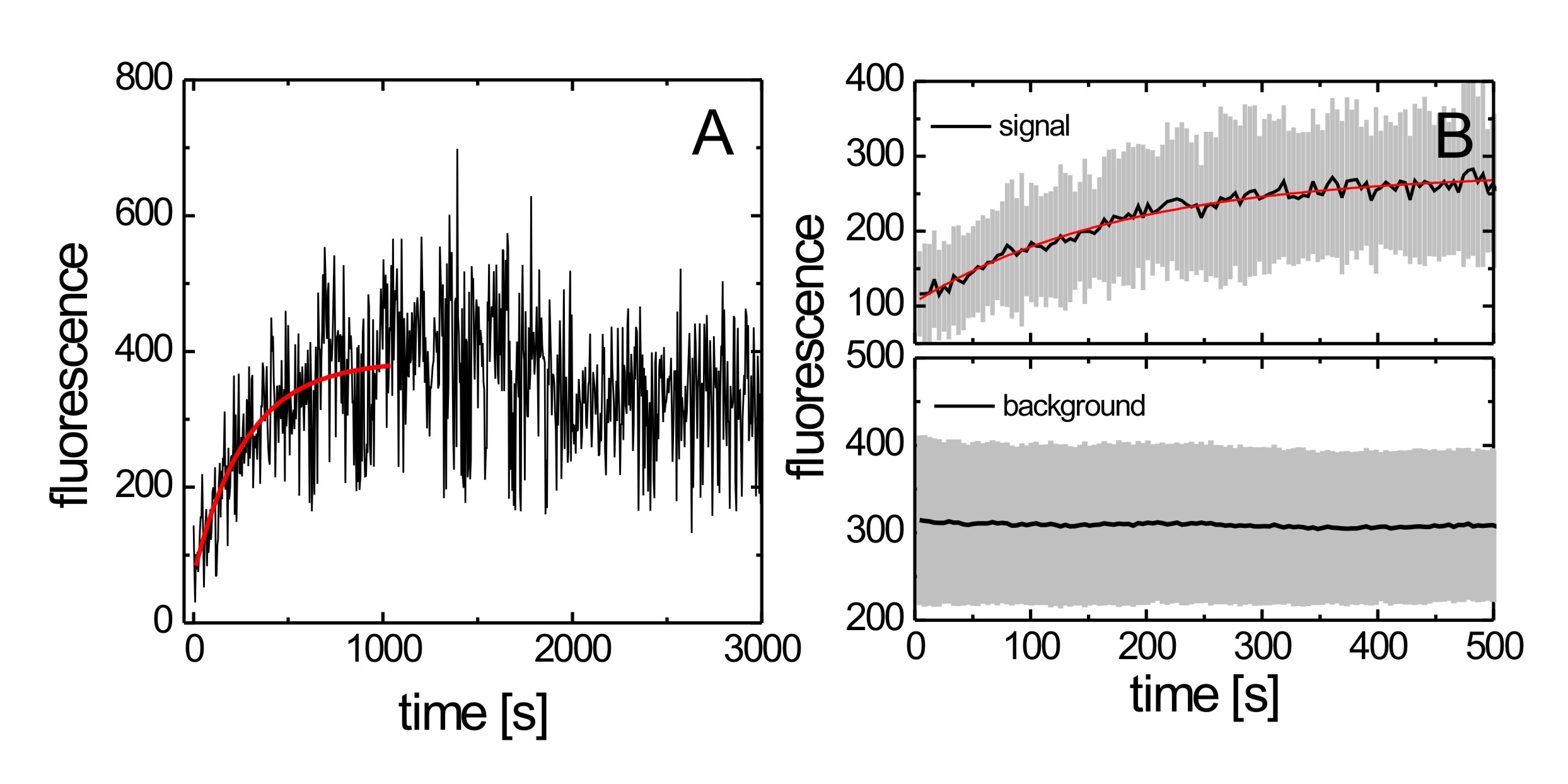

Supplement: Figure S4 — Assembly monitoring using SDCM. To ensure that the rates determined from TIRFM were not influenced by the strong dependence of the intensity of the distant of the fluorophores from the cover slip, additional experiments were performed using SCDM. The results of Phase I for HIVeGFP are shown for an individual assembly site (A) and for an average from 60 traces (B). The averaged value is shown in black along with the standard deviation of the data in grey and an exponential fit to the data in red. (0.33 MB JPG) [file ppat.1000652.s005.jpg]

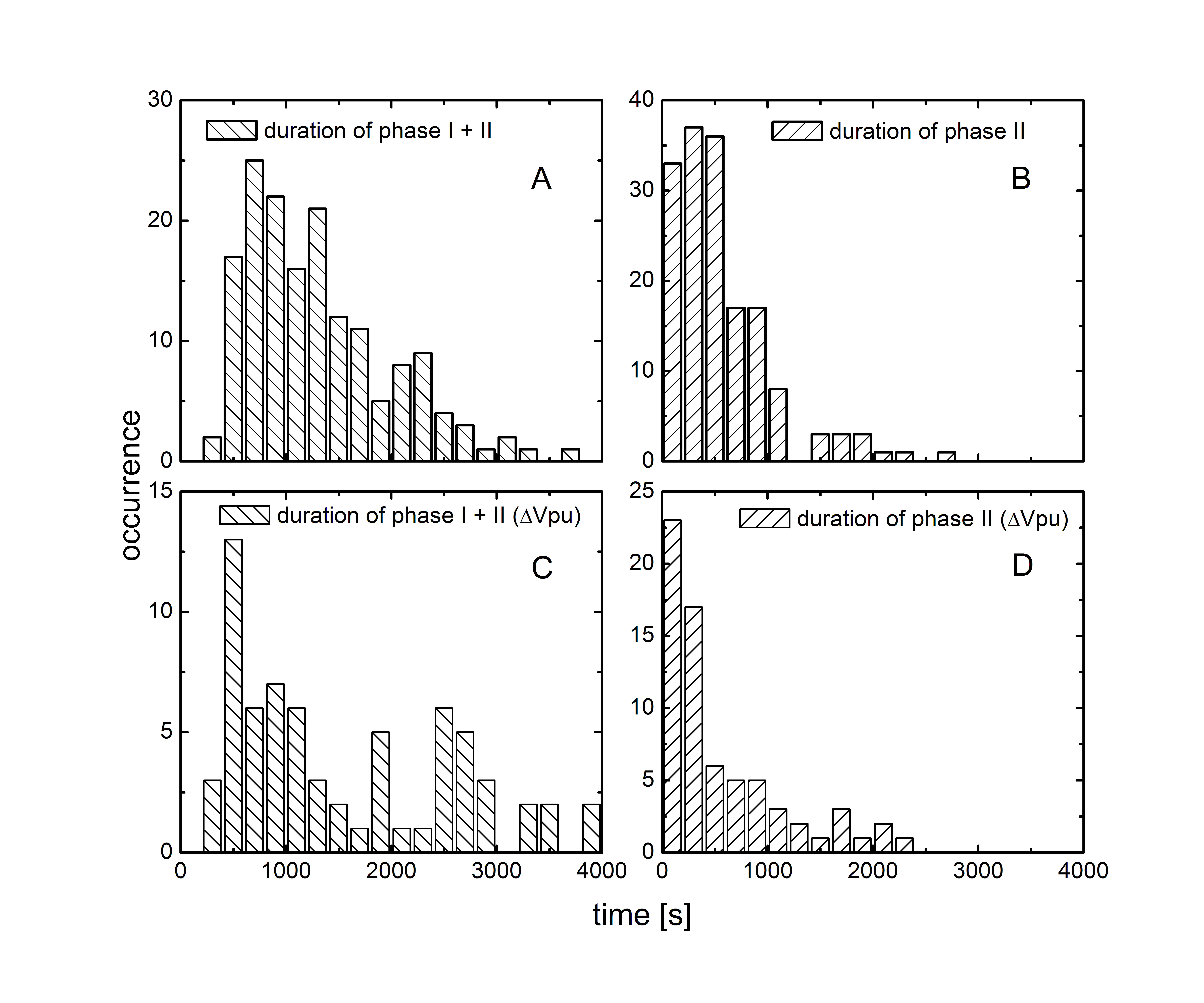

Supplement: Figure S5 — Histogram of the duration of phase I and II for HIV wild-type (A, B) and HIV ΔVpu (B,C) assembly. The time from the first appearance of the assembly site until the onset of phase III was determined for 160 (wt) and 69 (ΔVpu) individual assembly sites respectively (A, C). The duration of phase II was estimated as the time from 90% completion of phase I until the onset of phase III and is plotted in panels B and D for wt and ΔVpu cells respectively. A binning of 200 s was used for all of the histograms. (2.48 MB JPG) [file ppat.1000652.s006.jpg]

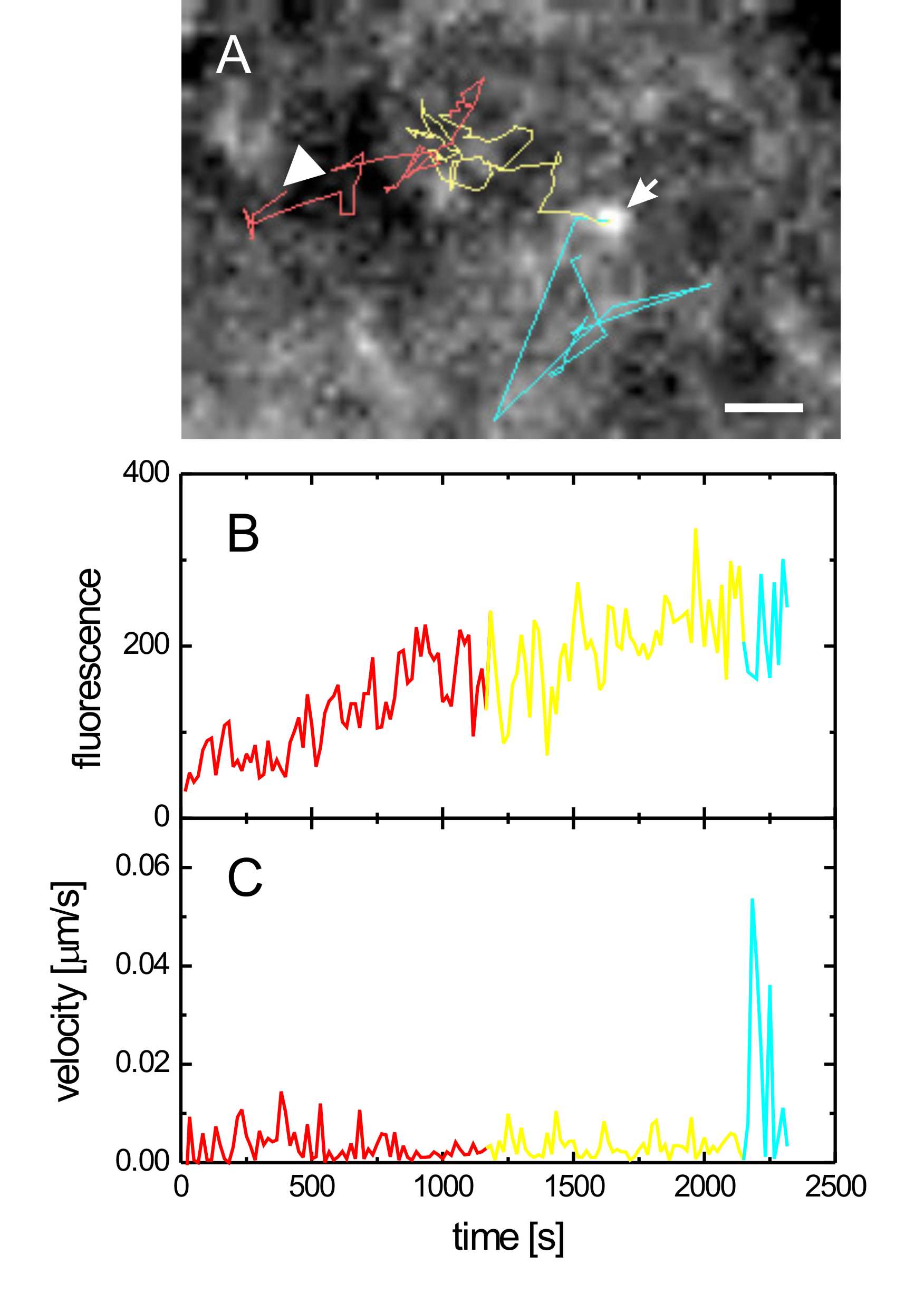

Supplement: Figure S6 — Release of HIV-1 observed using SDCM. (A) An image from Video S6 showing the assembly and release of an HI virion monitored on the dorsal membrane of the cell using SDCM (A). The trajectory, color coded to show the three phases, assembly phase (phase I, red), plateau region (phase II, yellow), and release (phase III, cyan) is overlaid. The beginning of the trajectory is marked with an arrowhead, while the arrow is pointing to a particle being followed. Scale bar = 1 µm. The fluorescence intensity (B) and instantaneous velocity (C) are shown for the above trajectory with the same color coding. (0.19 MB JPG) [file ppat.1000652.s007.jpg]

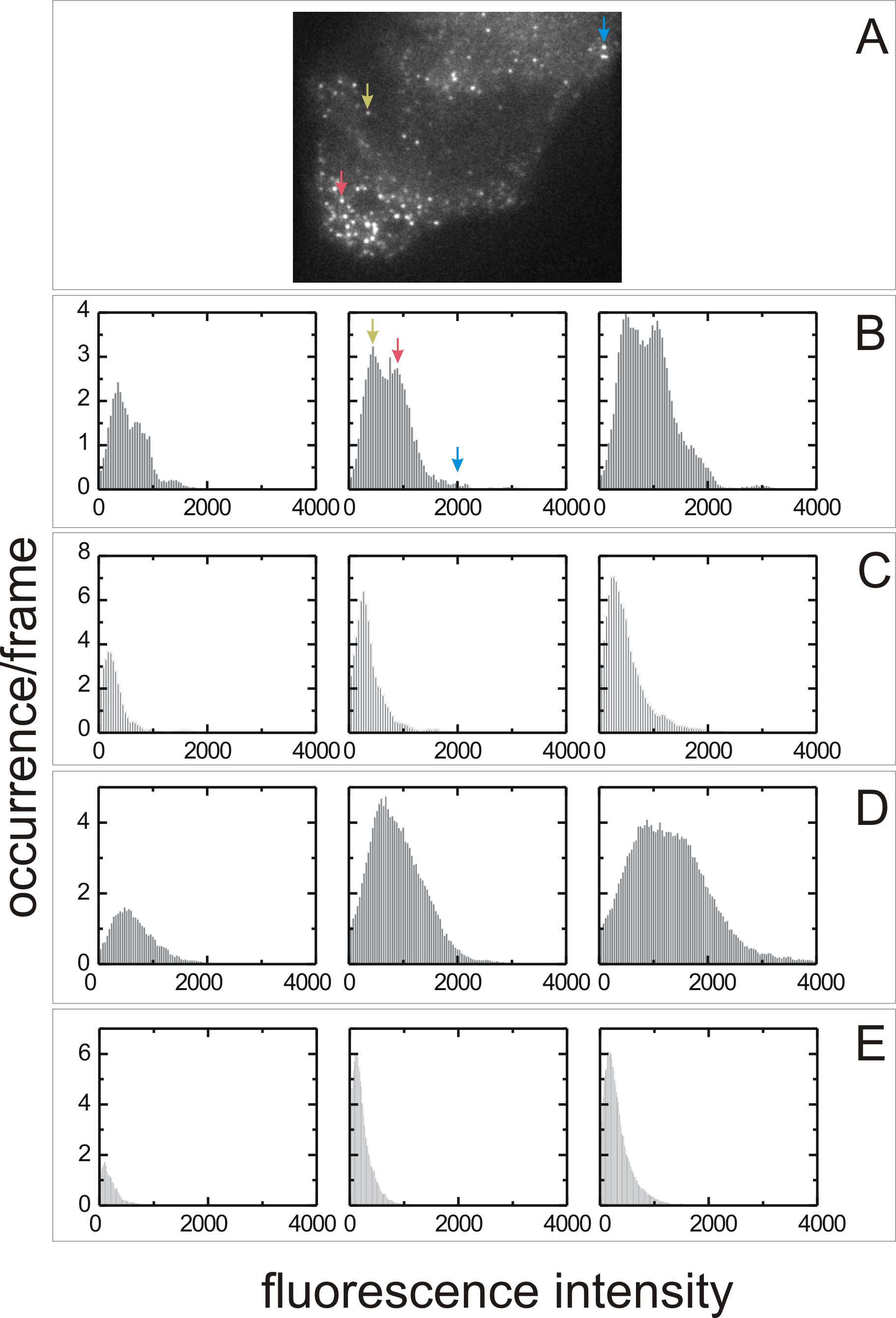

Supplement: Figure S7 — Fluorescence Intensity Distribution of Individual Assembly Sites. Image of a HeLa cell transfected with pCHIV/pCHIVeGFP imaged in TIRF mode (A); colored arrows indicate individual punctae that belong to different fluorescence intensity classes displayed in panel (B). Histograms of fluorescence intensities normalized per frame (B) for HIV/HIVeGFP in TIRF mode, (C) HIV/HIVeGFP in WF mode, (D) HIV(late-)/HIVeYFP(late-) in TIRF mode and (E) HIV(late-)/HIVeYFP(late-) in WF mode. Histograms correspond to 0–15 (left panels), 15–30 min (middle panels) and 30–45 min (right panels) after the start of data collection. As measurements were performed with different camera settings, the fluorescence intensities have been normalized to allow a direct comparison between experiments. (1.01 MB JPG) [file ppat.1000652.s008.jpg]

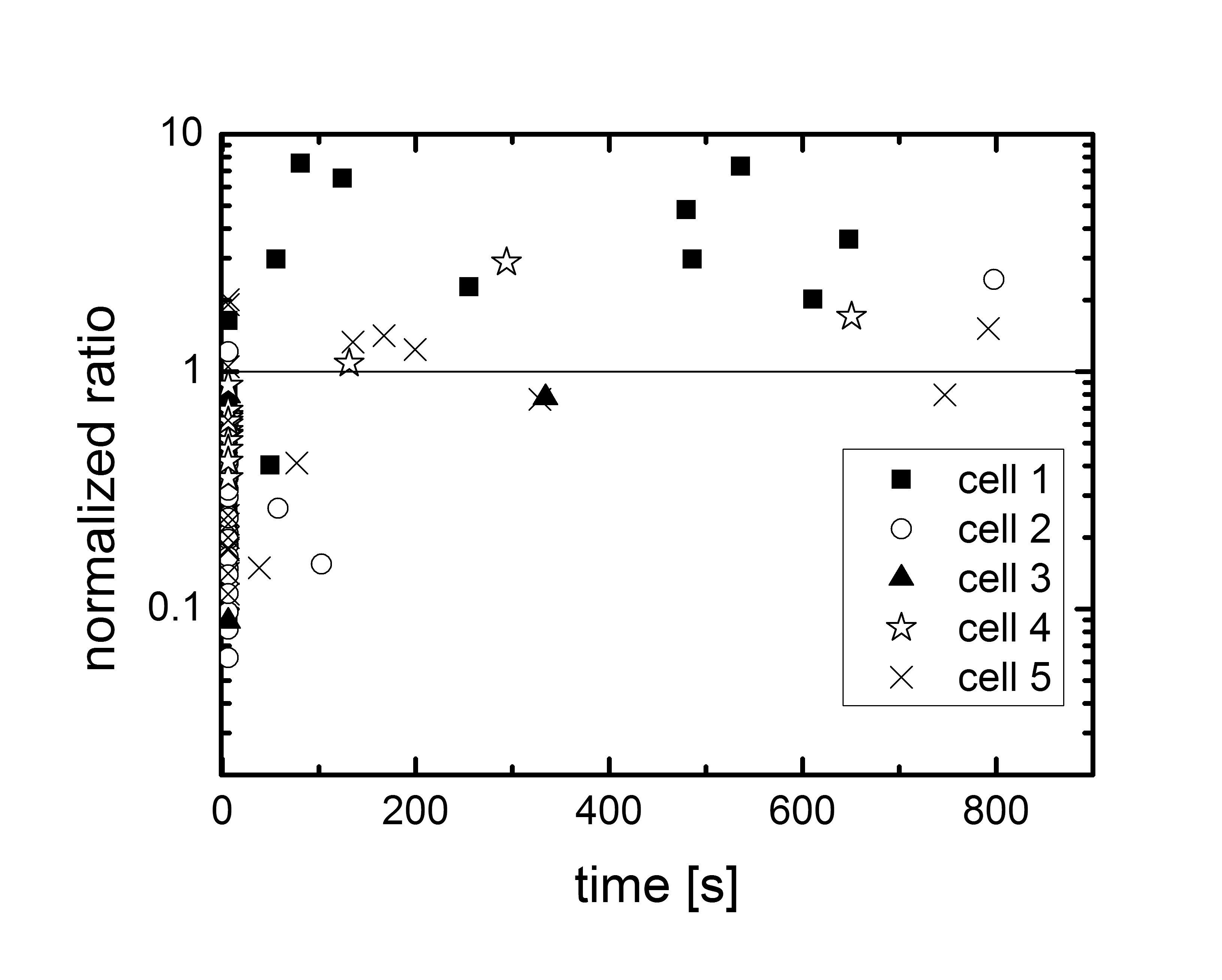

Supplement: Figure S8 — Recruitment of Gag after nucleation. The ratio of fluorescence intensity after green excitation to the fluorescence signal after red excitation normalized to the same ratio determined from the local background. Values are averaged over the time interval of 3 to 5 minutes after nucleation of the budding site. The normalized ratio is still greater than 1, indicating that the majority of Gag being incorporated into the budding at this time is recruited from the cytosol. (0.29 MB JPG) [file ppat.1000652.s009.jpg]

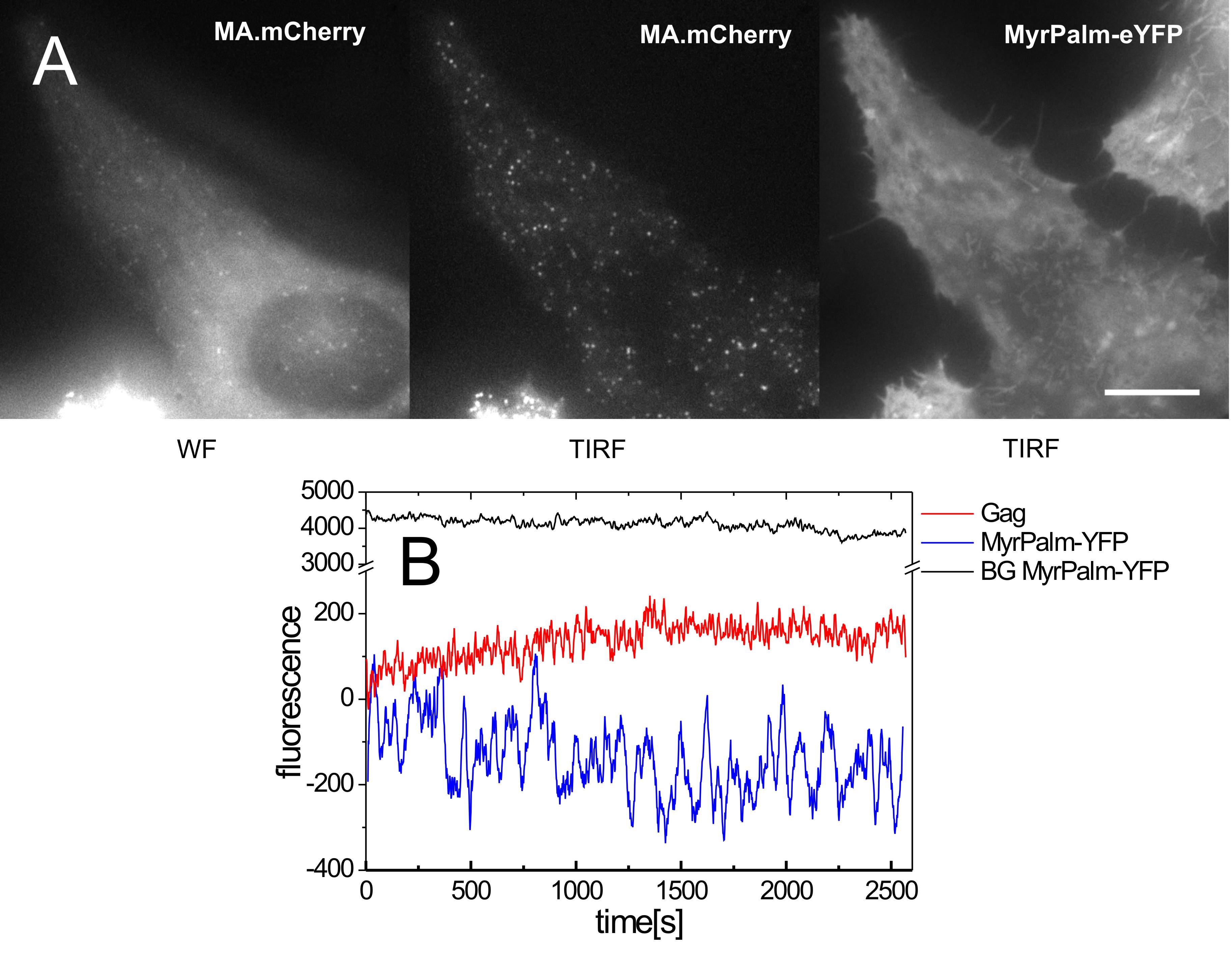

Supplement: Figure S9 — Comparison of the dynamics of MyrPalm-mYFP and HIV-1 Gag during viral assembly. (A) Images of mCherry-labeled Gag in wide field (left panel) and TIRF (middle panel) and MyrPalm-eYFP in TIRF (right panel) obtained at 30 hpt; the scale bar represents 10 µm (B) Fluorescence intensities of Gag.mCherry and MyrPalm-YFP as a function of time for an individual budding site. No concomitant accumulation of the MyrPalm.mYFP signal (shown in blue) with the Gag signal (show in red) was observed. (0.62 MB JPG) [file ppat.1000652.s010.jpg]
